# Rapid global antenna design by simplex regressors and multi-resolution simulations

**DOI:** 10.1038/s41598-025-96253-7

**Published:** 2025-04-04

**Authors:** Slawomir Koziel, Anna Pietrenko-Dabrowska, Stanislaw Szczepanski, Leifur Leiffson

**Affiliations:** 1 Engineering Optimization & Modeling Center, Reykjavik University, 102 Reykjavik, Iceland; 2 Faculty of Electronics, Telecommunications and Informatics, Gdansk University of Technology, Gdansk, 80 - 233 Poland; 3 School of Aeronautics and Astronautics, Purdue University, West Lafayette, IN 47907 USA

**Keywords:** Antennas, Design automation, Electromagnetic analysis, Characteristic points, Simplex, Variable-fidelity models, Engineering, Electrical and electronic engineering

## Abstract

Optimization methods have been rapidly entering the realm of antenna design over the last several years. Despite many available algorithms, practical optimization is demanding due to the high electromagnetic (EM) analysis cost necessary for dependable antenna assessment. This is particularly troublesome in global parameter tuning, routinely conducted using nature-inspired procedures. Unfortunately, these methods are known for their poor computational efficiency. Surrogate modeling may mitigate this issue to a certain extent, yet dimensionality and parameter range issues severely impede the construction of accurate metamodels. This research suggests an innovative algorithm for global parameter adjustment of antenna systems. It conducts a simplex-based search in the space of the structure’s performance figures (e.g., center frequencies, bandwidth, etc.). Operating at this level regularizes the objective function. Low cost is achieved by the simplex updating strategy requiring only one EM analysis per iteration, and multi-resolution simulations. The global search state involves coarse-discretization full-wave analysis, whereas final (gradient-based) parameter tuning involves medium-fidelity simulations for sensitivity estimation and high-fidelity models for design verification. The developed algorithmic framework is validated using four microstrip antennas. The results generated in multiple runs demonstrate global search capability and remarkably low expenses, corresponding to around a hundred high-fidelity analyses on average. The performance level is competitive over local and global optimizers.

## Introduction

Strict performance requirements generated by the needs of various fields of application (wireless communications^1,2^, internet of things^3^, space communications^4^, energy harvesting^5^, microwave imaging^6^, automotive radars^7^) make antenna design a challenging endeavor. It is further aggravated by functionality demands such as broadband^8^, multi-band^9^or MIMO operation^10^, tunability^11^, enhanced gain^12^, beam steering^13^, circular polarization^14^, as well as small physical dimensions^15–17^. Meeting these specifications leads to developing intricate topologies featuring large numbers of parameters. Their reliable evaluation requires EM analysis. Further, yielding the best possible design is contingent upon careful tuning of antenna dimensions while simultaneously controlling several responses and the antenna size. Traditionally used experience-driven parameter sweeping or utilization equivalent network models are no longer adequate for this purpose. Instead, rigorous optimization is suggested^18–20^, although it is computationally expensive, even in local search. Global algorithms^21–23^(including multi-criterial design routines^24–26^) incur significantly higher expenses. Yet, global search is required for a growing number of design scenarios that include multi-modal problems (e.g., design of coding metasurfaces^27^, frequency selective surfaces^28^, EM-driven miniaturization with performance constraints^29^, pattern synthesis of array antennas^30^, design of metamaterial lenses^31^), the lack of a good starting point^32^, or antenna scaling across extended ranges of operating frequencies and/or material parameters^33^.

Nowadays, global optimization is dominated by nature-inspired methods^34–37^. The traditional yet still widely used population-based approaches encompass genetic algorithms (GAs)^38^, evolutionary algorithms/strategies^39,40^, ant systems^41^, or genetic programming^42^. Some examples of newer methods are, among others, particle swarm optimization (PSO)^43^, differential evolution (DE)^44^, firefly algorithm^45^, or grey wolf optimization^46^. Recent years witnessed the development of vast amounts of algorithms (harmony search^47^, eagle strategy^48^, bacteria foraging optimization^49^, invasive weed optimization^50^, and many others^51–60^. The capability to perform global search stems from information exchange within the candidate solution set, either through recombination/mutation operators^61^, or through biasing the member relocation towards, e.g., local or population-wise best solution found so far^62^. Nature-inspired methods are straightforward to implement, and the operating flow is identical for most procedures^63^. The downside is unsatisfactory computational efficiency. As number of merit function calls during an optimization run ranges usually from hundreds to many thousands, direct antenna optimization with these algorithms is normally infeasible unless faster models are available (e.g., synthesis of a radiation pattern using analytical array factor models^64–66^), or EM analysis is relatively cheap (e.g., when antenna geometry is simple and coarse discretization is employed).

Global (especially nature-inspired) optimization of antennas is nowadays most often realized using surrogate modeling methods^24,67–70^. Popular modeling techniques include neural networks^71,72^, kriging^73^, support vector regression^74^, or Gaussian process regression^75^. Unfortunately, globally accurate models can only be constructed for simple structures. Consequently, surrogate-assisted procedures are typically in the form of machine learning frameworks^76,77^, where the surrogate is iteratively refined and used to predict candidate solutions^78^. Model refinement may target identifying a globally optimum design, but other infill criteria are also possible^79,80^. Optimization processes can also be expedited using physics-based surrogates^81–83^, typically used for local search.

Other challenges in constructing reliable data-driven antenna models include response nonlinearity and the need to model several responses (reflection, gain, axial ratio) over broad frequency ranges. Accordingly, many surrogate-based techniques are only demonstrated using structures operating over limited parameter spaces^84,85^. The recently reported performance-driven modeling techniques^86–89^allow for mitigating some of these issues by focusing on regions with high-quality designs. A performance-driven modeling paradigm has been employed for general-purpose meta-modeling^90^, but it has also expedited multi-objective optimization^91^and robust design^92^. Yet another approach is the response feature technology^93,94^, which capitalizes on restating the task using adequately determined characteristic (or feature) points, e.g., resonances, local maxima/minima of reflection or gain within the operating bandwidth, etc. This regularizes the merit function, enables the acceleration of optimization processes^95^, and enhances the computational efficiency of surrogate model rendition^96^.

This research suggests a new algorithm for the global design of antennas. The major prerequisites are reliability and computational efficiency. Both are enabled by arranging the optimization process as a simplex-based search constructed regarding operating figures obtained from EM simulations, rather than the complete frequency characteristics. This allows for achieving the benefits inherent to the response-feature approaches (objective function landscape regularization), which facilitates finding the optimum but also expedites the process. The two additional factors contributing to the algorithm’s efficiency are the strategy of updating the simplex and the incorporation of multi-resolution simulations. The early optimization stages employ coarse-discretization EM analysis, whereas final (gradient-based) tuning is executed using mixed models: high-fidelity for design verification and medium-fidelity for sensitivity estimation. Furthermore, the implemented mechanism of reducing the simplex size guarantees convergence. A comprehensive verification of our methodology uses four microstrip antennas of different characteristics. The numerical results indicate its superior performance over the benchmark concerning the ability to render high-quality designs consistently (i.e., for all performed runs), repeatability of results, and computational efficiency. The average CPU expenses of the search process amount to only 95 high-fidelity EM antenna analyses. While this low-cost results from the overall algorithm architecture, the additional acceleration factor resulting from multi-resolution models is as high as 1.3 on average, as estimated by comparison with the high-fidelity simplex-based search.

The technical contributions of this work include: (i) the development of antenna optimization framework with global search capability achieved using simplex regressors constructed for the operating parameters, (ii) incorporation of efficient design relocation strategy and variable-resolution EM simulations to guarantee cost efficiency, (iii) development of mechanisms that guarantee a formal convergence, (iv) implementation of the algorithmic framework combining global and rapid local tuning stages, (v) conclusive demonstration of the efficacy of the introduced methodology both regarding reliability, final design quality, and low running cost.

## Global optimization with simplex regressors and Variable-Resolution models

This part of the work provides the details of the presented optimization strategy. The underlying concept is the employment of simplex regressors representing the antenna’s operating parameters. By exploiting regular dependence between antenna geometry and operating parameters, a rapid determination of the promising region becomes possible. The process is accelerated even further by the utilization of low-resolution EM analysis. To ensure reliability, global search is complemented by final tuning at the high-resolution level.

### EM-Driven optimization

The problem is defined using the design objectives and constraints and the type of responses to be handled. The antenna adjustable parameters are marked as ***x*** = [*x*_1_ … *x*_*n*_]^*T*^. In contrast, target operating frequencies are represented as ***f***_*t*_ = [*f*_*t*.1_ … *f*_*t*.*K*_]^*T*^, assuming a *K*-band structure. The vector ***x*** is assessed using a merit function *U*(***x***,***f***_*t*_). It is established so that smaller values of *U* correspond to better designs. The optimum solution x^*^ is rendered as

1 $${x}^*=\arg \mathop {\hbox{min}}\limits_{x} U(x,f_t)$$

**Fig. 1:**
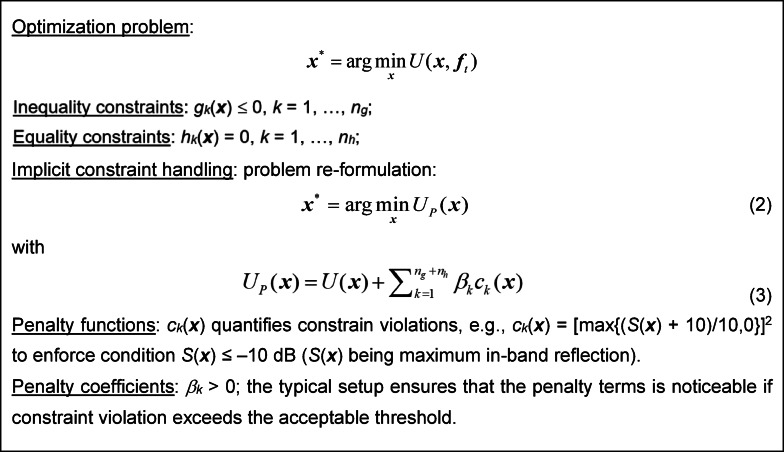
Constrained optimization and the outline of constraint handling in an implicit manner using penalty functions.

The problem (1) may be constrained. If constraint evaluation requires EM analysis, a convenient handling approach is through penalty functions^105^, as outlined in Fig. 1.

In formulation (1), the operating frequencies are explicitly distinguished to emphasize that their proper placement is the fundamental challenge in optimizing high-frequency systems. Also, the algorithm introduced here relies on metrics quantifying the discrepancy between the actual and target frequencies. Hence, this notation is necessary for further consideration.

### Multi-Resolution EM analysis

The concept of multi-resolution modeling has been explored in high-frequency design since the early 2020 s^97^. For antennas, a flexible approach to rendering lower-fidelity representation is the reduction of the structure’s discretization in the EM analysis process and introducing other simplifications (e.g., neglecting losses, etc^98^).

**Fig. 2:**
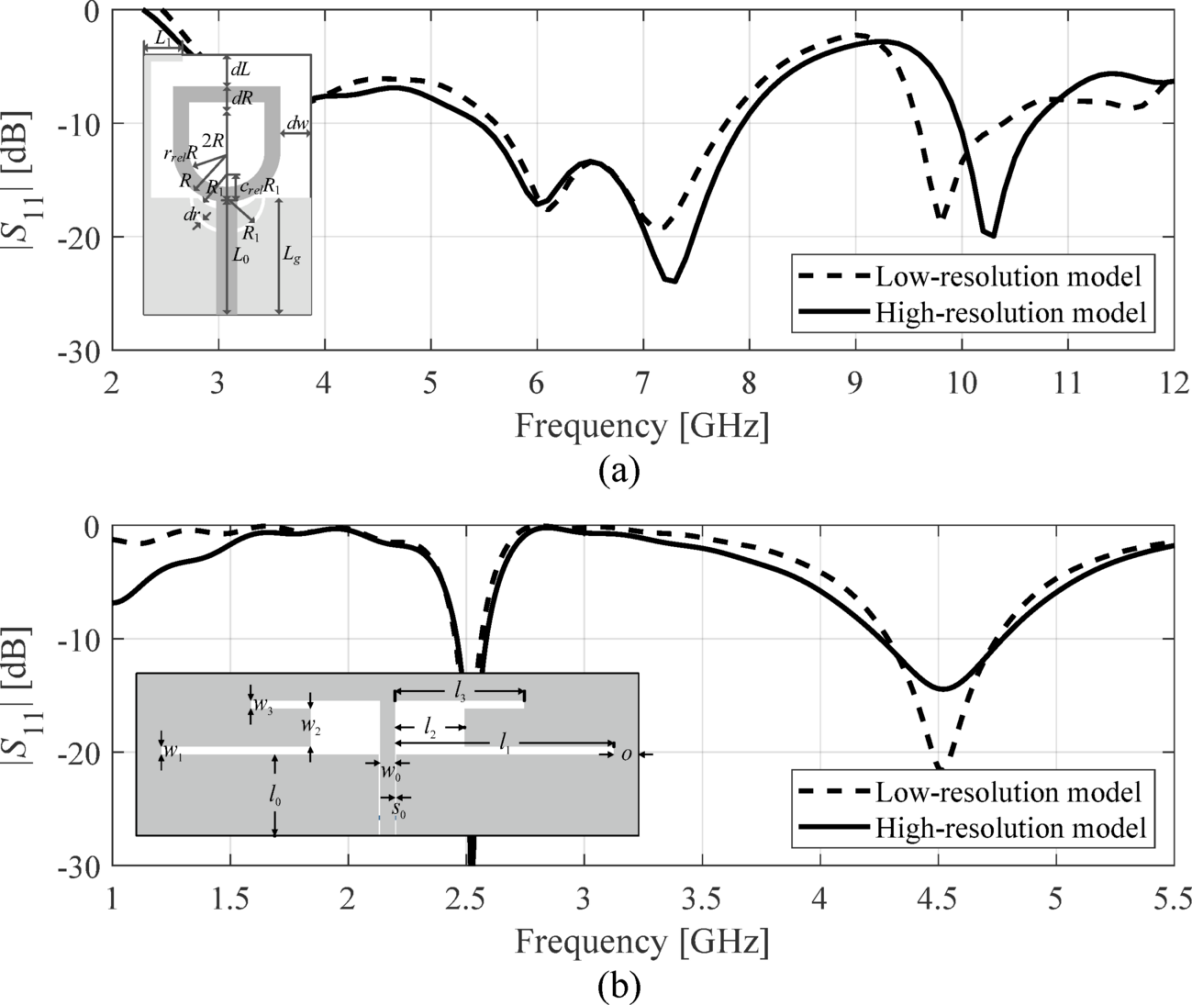
Responses of simulation models of two fidelities (low-fidelity ***R***_*c*_ (- - -) and fine model ***R***_*f*_ (—)): (**a**) a miniaturized broadband monopole antenna (the model includes the SMA connector), the simulation times for ***R***_*f*_ and ***R***_*c*_ are about 400 s and 50 s, respectively; (**b**) a dual-band antenna, the ***R***_*f*_ and ***R***_*c*_ simulation times are about 90 s and 25 s, respectively.

Reducing the resolution of the simulation process allows for shortening the analysis time while compromising the dependability^99^, cf. Figure 2. The achievable speedup is typically from three to ten, assuming the low-fidelity analysis accounts for relevant antenna characteristic features. In practice, two levels of resolution are employed: low (coarse discretization) and high (fine model)^100^, although more sophisticated model management schemes have been developed as well^101^, including continuous adjustment of the model fidelity^102^. For specific applications, e.g., local optimization with space mapping^103^, it is mandatory to enhance the low-fidelity model, but it may be applied as is for other purposes, e.g., pre-screening^104^.

Here, we use two models: low-resolution ***R***_*c*_(***x***) and high-resolution ***R***_*f*_(***x***). ***R***_*c*_(***x***) is used in the global search stage (cf. Sects. Simplex-Based regression models and Global search). The high-resolution model will be employed for the final tuning of antenna parameters, cf. Sect. Final tuning.

### Simplex-Based regression models

The performance of antenna structures is contingent upon the appropriate parameter adjustment. The high cost incurred by multiple EM simulations involved in the optimization process, even for local tuning, is a serious obstacle. In a growing number of cases, global search is necessary, which is hindered by several factors that include the need for exploring vast spaces, response nonlinearity and considerable relocations of the operating frequencies.

**Fig. 3:**
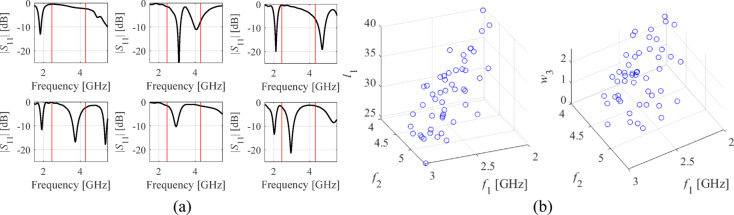
Relationship between the entire antenna characteristics and its geometry parameters versus analogous relationship for antenna operating parameters: (**a**) |*S*_11_| of a dual-band antenna form Fig. 2(b) at several trial points; (**b**) resonant frequencies *f*_1_ and *f*_2_ versus two (out of six) geometry parameters of the same antenna (random designs). The dependencies shown in panel (b) are clearly more linear than those presented in panel (a).

Inherent difficulties of EM-driven design can be mitigated using surrogate-assisted approaches^24,67–83^. However, rendering accurate behavioral models over large spaces is generally infeasible. Consider Fig. 3(a), showing |*S*_11_| of a dual-band antenna at random designs. Local optimization originating from most of the shown designs will be unsuccessful in moving the operating frequencies toward the required values. Meanwhile, constructing an accurate data-driven model of highly nonlinear characteristics such as those in Fig. 3(a) requires large training datasets.

The same situation examined from the standpoint of antenna operating parameters gives a different picture. The relationships between the resonant frequencies *f*_1_ and *f*_2_ and antenna geometry parameters, cf. Figure 3(b), are simpler and essentially monotonic, even though the plots were obtained using random observables. The literature on feature-based modeling concurs that these dependencies are common for high-frequency structures^93–96^.

**Fig. 4:**
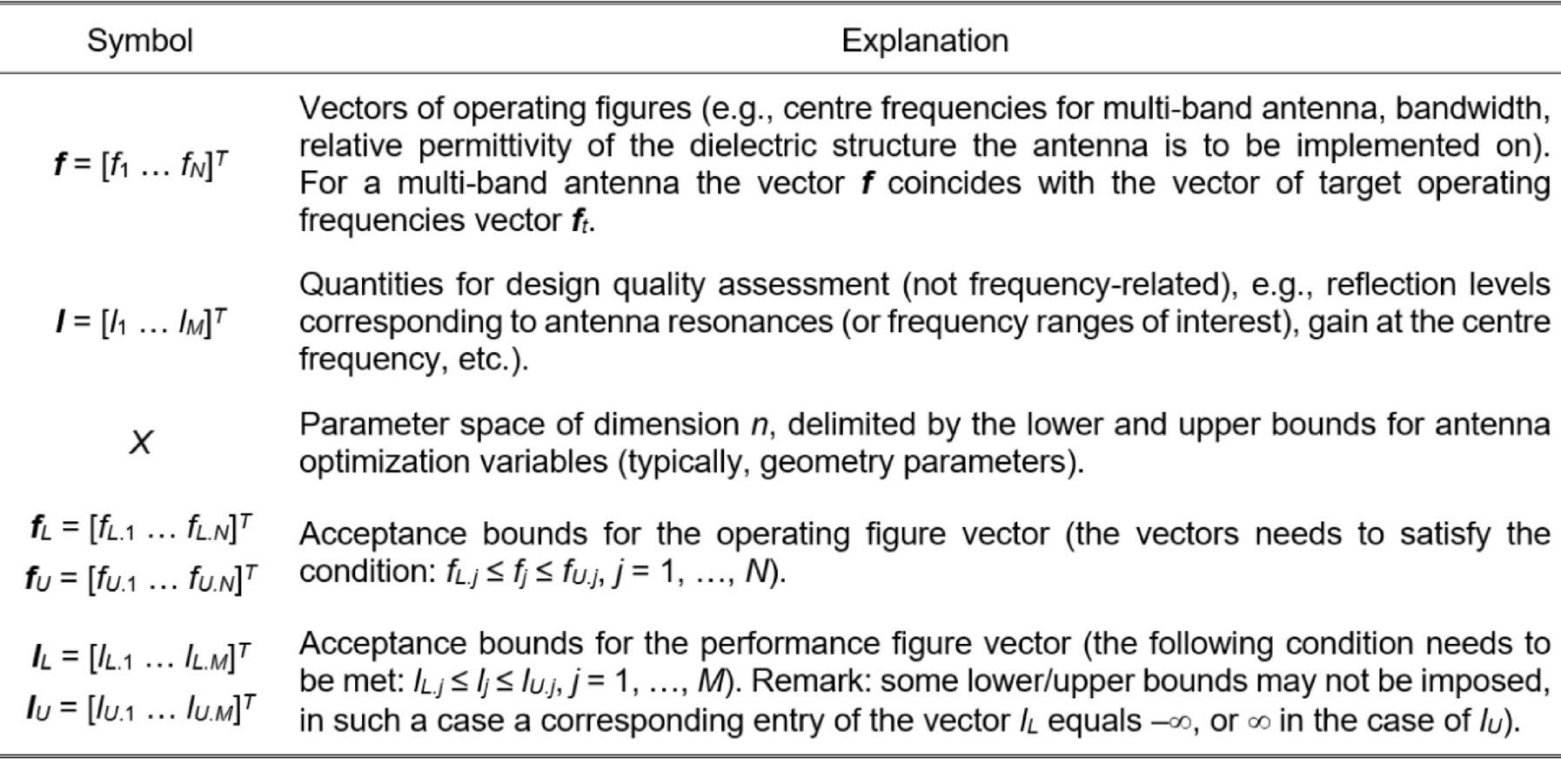
Notation used in the context of simplex-based regression models.

The algorithm introduced in this work is developed to explore the relationships illustrated in Fig. 3. The simplicity of the mentioned dependencies allows us to conduct globalized optimization based on structurally uninvolved surrogate models, which are defined using the operating parameters instead of the original antenna outputs. At the same time, whatever model is used, it must reflect the full dimensionality *n* of the parameter space. This implies incorporating no less than *n* + 1 affinely independent points ***x***^(*j*)^. The most straightforward object fulfilling these requirements is a simplex, which is also suitable for handling the relationships demonstrated in Fig. 3. Below, we provide a formal definition of a simplex-based regression model utilized in this work and elaborate on its use for antenna optimization. The relevant notation has been introduced in Fig. 4.

Consider affinely independent points ***x***^(*j*)^ = [*x*_1_^(*j*)^ … *x*_*n*_^(*j*)^]^*T*^, *j* = 0, …, *n*, in the parameter space *X* (*n* + 1 points in total). The pertinent operating figure vectors and performance figure vectors are ***f***^(*j*)^ = ***f***(***x***^(*j*)^) = [*f*_1_^(*j*)^ … *f*_*N*_^(*j*)^]^*T*^ and ***l***^(*j*)^ = ***l***(***x***^(*j*)^) = [*l*_1_^(*j*)^ … *l*_*M*_^(*j*)^]^*T*^, respectively. All points ***x***^(*j*)^ reside within acceptance bounds ***f***_*L*_ ≤ ***f***^(*j*)^ ≤ ***f***_*U*_, and ***l***_*L*_ ≤ ***l***^(*j*)^ ≤ ***l***_*U*_, for *j* = 0, …, *n*. Identification {***x***^(*j*)^}_*j* =0, …, *n*_, is realized through random sampling.

The EM-simulated responses at these points subsequently undergo an extraction process that permits assessing the respective operating and performance parameters ***f***(***x***^(*j*)^) and ***l***(***x***^(*j*)^). Only parameter vectors exhibiting distinct resonances within the specified ranges are considered suitable for predictor construction.

Given {***x***^(*j*)^}_*j* =0, …, *n*_, we can define the simplex-based regression models. As ***x***^(*j*)^ – ***x***^(0)^ are linearly independent, any parameter vector ***x*** ∈ *X* can be written as

4 $$x={x^{(0)}}+\sum\limits_{{j=1}}^{n} {{a_j}} ({x^{(j)}} - {x^{(0)}}),{\text{ with }}a(x)={X^{ - 1}}(x - {x^{(0)}})$$

In (5), the non-singular *n* × *n* matrix ***X*** is assembled as

5 $$X=[{{{x}}^{(1)}} - {{{x}}^{(0)}} \ldots {{{x}}^{(n)}} - {{{x}}^{(0)}}]$$

The performance and operating vectors are rendered using the regression models ***F***(***x***) : *X* → *F*, and ***L***(***x***) : *X* → *R*^*M*^, respectively, as

6 $$F(x)={f^{(0)}}+\sum\limits_{{j=1}}^{n} {{a_j}} ({f^{(j)}} - {f^{(0)}})={f^{(0)}}+{X_f}a(x)={f^{(0)}}+{X_f}{X^{ - 1}}(x - {x^{(0)}})$$

7 $$L(x)={I^{(0)}}+\sum\limits_{{j=1}}^{n} {{a_j}} ({I^{(j)}} - {I^{(0)}})={I^{(0)}}+{X_j}a(x)={I^{(0)}}+{X_j}{X^{ - 1}}(x - {x^{(0)}}){\text{ }}$$

The coefficient vectors ***a*** are obtained using (5), whereas the matrices ***X***_*f*_ and ***X***_*l*_ are defined as.

8 $${{\text{X}}_f}=[{f^{(1)}} - {f^{(0)}} \ldots {f^{(n)}} - {f^{(0)}}],\quad {{\text{X}}_I}=[{I^{(1)}} - {I^{(0)}} \ldots {I^{(n)}} - {I^{(0)}}]{\text{ }}$$

### Global search

The global search stage will be conducted using the regression models of Sect. Simplex-Based regression models using the low-resolution EM model ***R***_*c*_. As the operating parameters and geometry variables are in a weakly nonlinear relationship, ***F***(***x***) and ***L***(***x***) are expected to predict antenna performance in the space *X*, especially near the vector set {***x***^(*j*)^}_*j* =0, …, *n*_.

#### Evaluating design quality

Any optimization procedure requires an appropriate assessment of design quality. Here, it is realized using a scalar function *U*_*F*_, which assesses the antenna based on its operating and performance vectors ***f***(***x***) and ***l***(***x***). The aim is to align the operating vector ***f***(***x***) with the target ***f***_*t*_. To simplify notation, ***f***_*t*_ stands for the target operating figure vector and the target operating frequency vector (cf. Sect. EM-Driven optimization), although they are formally different. Yet, in all verification examples of Sect. Algorithm verification and benchmarking, the two vectors are identical.

The objective function *U*_*F*_ is

9 $${U_F}(x)=U(f(x),l(x))={U_L}(l(x))+{\beta _F}{\left\| {f(x) - {f_r}} \right\|^2}$$

In (9), *U*_*L*_ is similar to *U* but it is computed using the performance vector ***l***(***x***) instead of the complete antenna outputs. For clarification, consider the following examples:

##### • Example 1

**Fig. 5:**
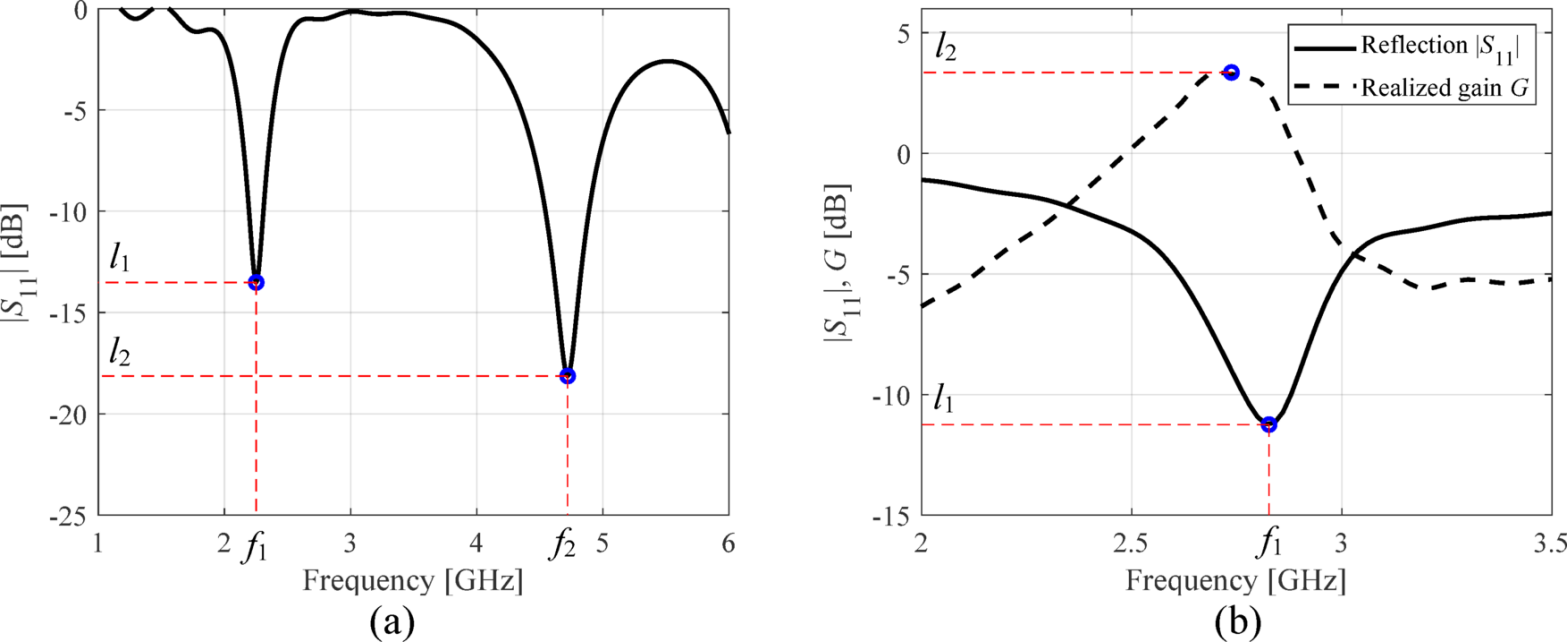
Operating and performance parameters: (**a**) dual-band antenna of Fig. 2(b) (***f*** = [*f*_*1*_ *f*_*2*_]^T^ – resonant frequencies; ***l*** = [*l*_*1*_ *l*_*2*_]^T^ – reflection at *f*_*1*_ and *f*_*2*_) (**b**) quasi-Yagi antenna (cf. Sect. Algorithm verification and benchmarking) (***f*** = *f*_*1*_, ***l*** = [*l*_*1*_ *l*_*2*_]^T^ – reflection at *f*_*1*_ and gain at its maximum).

• Let ***l***(***x***) = [*l*_1_ … *l*_*M*_]^*T*^ stand for |*S*_11_| at *M* resonances of an antenna. To improve reflection at operating frequencies *f*_1_ through *f*_*M*_, one may define *U*_*L*_(***l***(***x***)) = max{*l*_1_,…,*l*_*M*_} (cf. Figure 5(a)).

##### • Example 2

• Let ***l*** *=* [*l*_1_ *l*_2_]^*T*^ be a performance vector of the quasi-Yagi antenna (cf. Figure 5(b)), with *l*_1_ being the reflection level at antenna operating frequency *f*_1_, and *l*_2_ being the maximum realized gain. If the goal is to enhance the gain at *f*_1_, we define *U*_*L*_(***l***(***x***)) = –*l*_2_.

Figure 5 shows the examples of vectors ***f*** and ***l*** for a dual-band and quasi-Yagi antennas.

Note that *U*_*L*_ does not need to exactly mimic *U*(***x***), which is particularly the case when responses are supposed to be controlled at specific bandwidths. The main concern of global search is to bring ***f***(***x***) possibly close to ***f***_*t*_. The main role is played by the regularization term in (9), with *β*_*F*_ being the enforcement factor.

#### Simplex refinement

The information on antenna operating and performance figures is encoded in ***F***(***x***) and ***L***(***x***). We use it to identify a location of the optimum according to function *U*_*F*_ of (9). We have

10 $${{\text{x}}_{tmp}}=\arg {\hbox{min} _{x \in X}}{U_F}(F({\text{x}}),L({\text{x}}))$$

where ***f***(***x***) and ***l***(***x***) are predicted by ***F***(***x***) and ***L***(***x***). As the regression models are the most accurate close to the vertices {***x***^(*j*)^}_*j* =0,…,*n*_, the process (10) is restricted to a small neighborhood of the simplex. Solving (10) is subject to constraints defined using ***a***(***x***) of (4)

11 $$\sum\limits_{{j=1}}^{n} {{a_j}} =1$$

12 $$- \alpha \leqslant {a_j} \leqslant 1+\alpha ,\quad j=1, \ldots ,n$$

Here, *α* > 0 is a small number (e.g., *α* = 0.2).

**Fig. 6:**
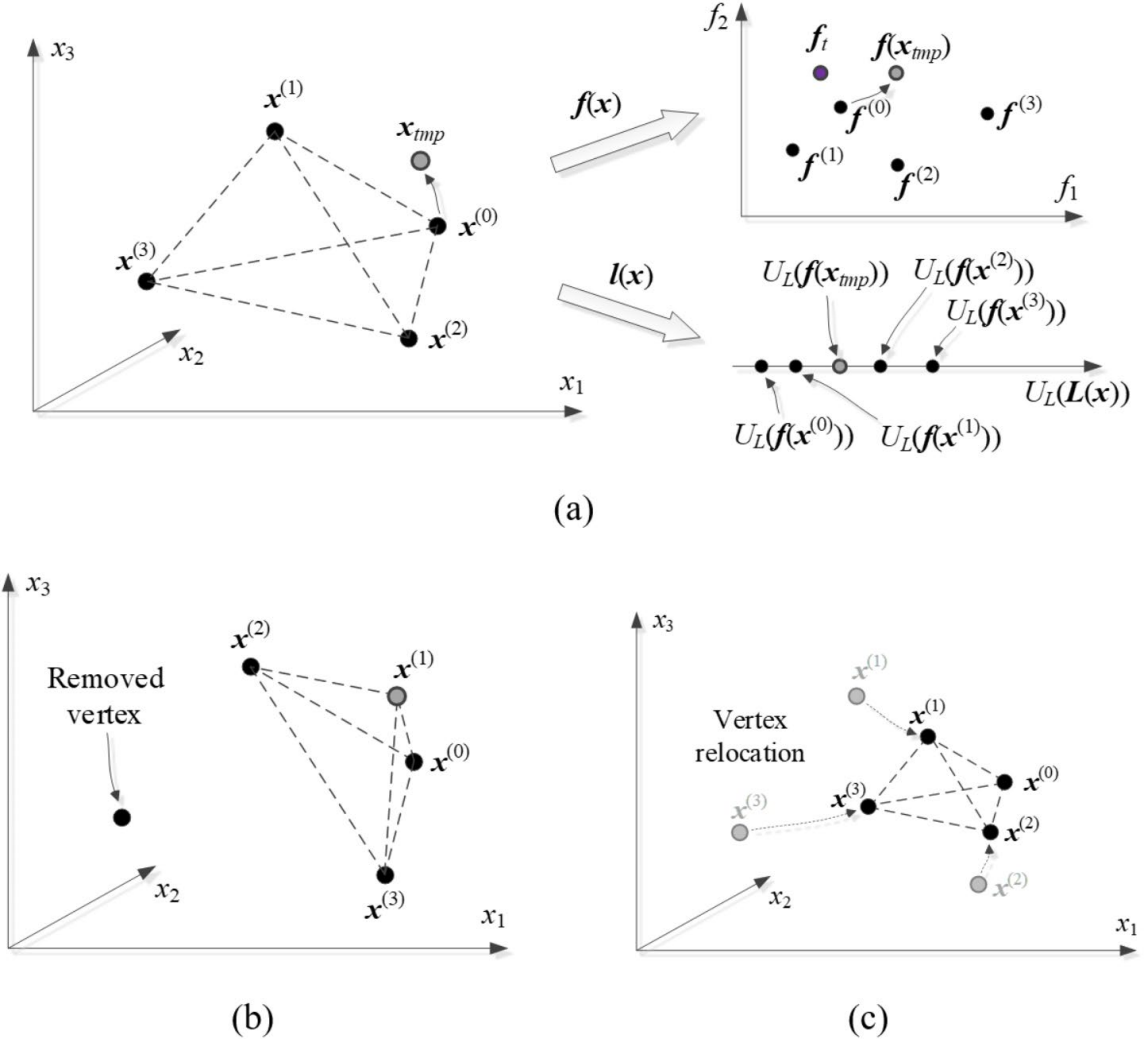
Global search: (**a**) current simplex. The predictions obtained using models ***F***(***x***) and ***L***(***x***) are juxtaposed against ***f***(***x***) and ***l***(***x***) at ***x***_*tmp*_ obtained by (10). Here, ***x***_*tmp*_ will be accepted as it improves *U*_*L*_ and ||***f***(***x***) – ***f***_*t*_||; (**b**) corresponding simplex update (illustrated assuming that ***x***_*tmp*_ is better than ***x***^(1)^ but inferior to ***x***^(0)^); (**c**) simplex reduction if ***x***_*tmp*_ was rejected.

In subsequent considerations, we order the vertices regarding ||***f***^(*j*)^ – ***f***_*t*_||. Thus, ***x***^(0)^ corresponds to the smallest distance between ***f***(*j*) and ***f***_*t*_. For the same reason, solving (10) starts from ***x***^(0)^, which is the best available design in the alignment between the actual and target parameters. Observe that ***a***(***x***^(0)^) = [0 … 0]^*T*^. The process of generating the candidate design is shown in Fig. 6. ***x***_*tmp*_ is only accepted if it improves the simplex quality, that is, if

13 $$\left\| {{f_{tmp}} - {f_t}} \right\|<\hbox{max} \left\{ {j \in \left\{ {0,1, \ldots ,n} \right\}:\left\| {{f^{(j)}} - {f_t}} \right\|} \right\}$$

where ***f***_*tmp*_ = ***f***(***x***_*tmp*_). If (13) holds, then ***x***_*tmp*_ replaces ***x***^(*jworst*)^, in which

14 $${j_{{\text{worst}}}}=\operatorname{argmax} \{ j \in \{ 0,1, \ldots ,n\} :\left\| {{f^{(j)}} - f} \right\|\}$$

In case of rejecting ***x***_*tmp*_, the simplex is reduced towards ***x***^(0)^ as

15 $${{\text{x}}^{(j)}} \leftarrow \gamma {{\text{x}}^{(j)}}+(1 - \gamma ){{\text{x}}^{(0)}}\quad {\text{ for }}j=1, \ldots ,n{\text{ }}$$

Therein, the reduction coefficient is established at *γ* = 0.5 so that the reduction is carried out to half of the original size. It can be shown (details omitted here) that under mild conditions on ***f***(***x***) (specifically, its continuous differentiability), sufficient reduction of the simplex size improves vertex quality by reducing the corresponding norms ||***f***^(*j*)^ – ***f***_*t*_||.

The simplex updating procedure is terminated if any of the following conditions holds:

- *Operating parameter convergence*:

16 $$||{\mathbf{f}}({{\mathbf{x}}_{tmp}}) - {{\mathbf{f}}_t}|| \leq {F_{\hbox{max} }}$$

Here, the control parameter *F*_max_ is set up in a relaxed manner, only to ensure that the optimum is reachable through local tuning. In practice, *F*_max_ should be equal to a fraction (twenty to 50%) of the expected bandwidths.

- *Exceeding computational budget*: the number of EM analyses exceeds $N_{global}$ (control parameter);
- *Sufficient simplex reduction*: $D= max {\{j \in \{1, 2, ...,n}\}: \Vert x^{(j)}-x^{(0)}\Vert\}< D_{min}$ (control parameter).

The last two conditions ensure convergence even if (16) cannot be fulfilled.

### Final tuning

The global search stage is concluded upon finding the parameter vector ***x***^(0)^ that satisfies the condition (18) (or any of the two convergence-related criteria, cf. Sect. Simplex refinement). The next stage is final parameter tuning involving a trust-region (TR) gradient-algorithm^106^. It is a gradient-based procedure with response sensitivities evaluated using finite differentiation (FD)^107^. The algorithm is terminated if the distance between subsequent iteration points ||***x***^(*i*+1)^ – ***x***^(*i*)^|| < *ε*(control parameter). To improve the efficiency, FD is replaced by a rank-one Broyden formula^108,109^ if ||***x***^(*i*+1)^ – ***x***^(*i*)^|| < *M*_*c*_*ε*, where *M*_*c*_ = 10. For reliability, the local tuning is carried out using ***R***_*f*_.

### Optimization procedure

The proposed framework combines the components discussed in Sects. Multi-resolution EM analysis through Final tuning. The input data includes the parameter space *X*, EM models ***R***_*c*_ and ***R***_*f*_, definitions of the operating vector ***f*** and performance vector ***l***, the target operating parameter vector ***f***_*t*_, as well as the analytical formulation of the objective function *U*_*L*_ (cf. (11)). Note that *X*, ***f***, ***l***, ***R***_*c*_, and ***R***_*f*_, are antenna-dependent, whereas ***f***_*t*_ and *U*_*L*_ are determined by design specifications.

**Fig. 7:**
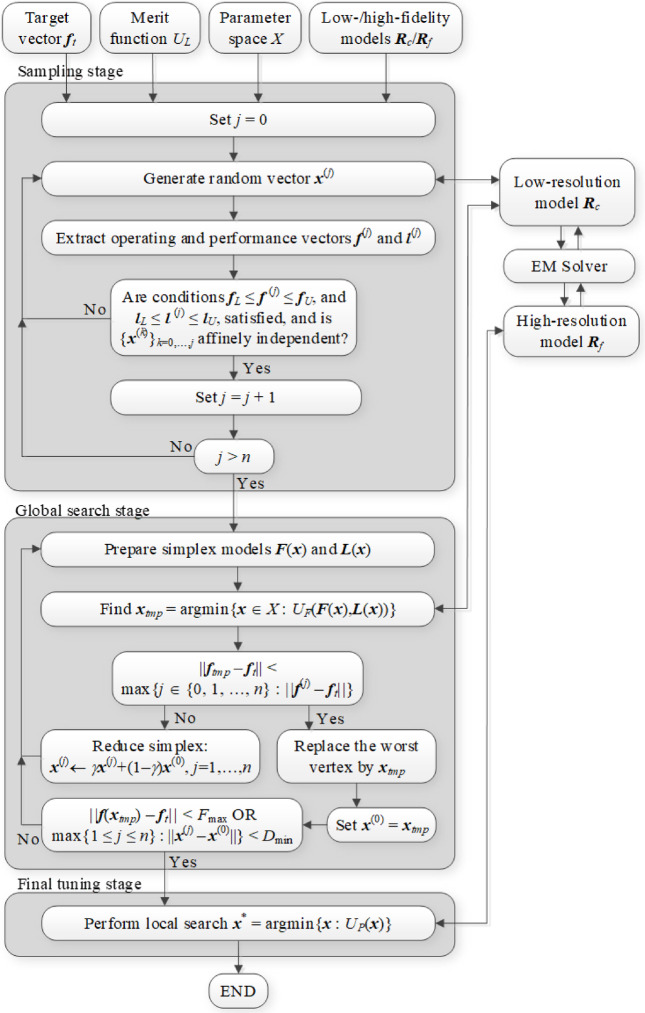
Proposed optimization procedure: flow diagram.

**Fig. 8:**
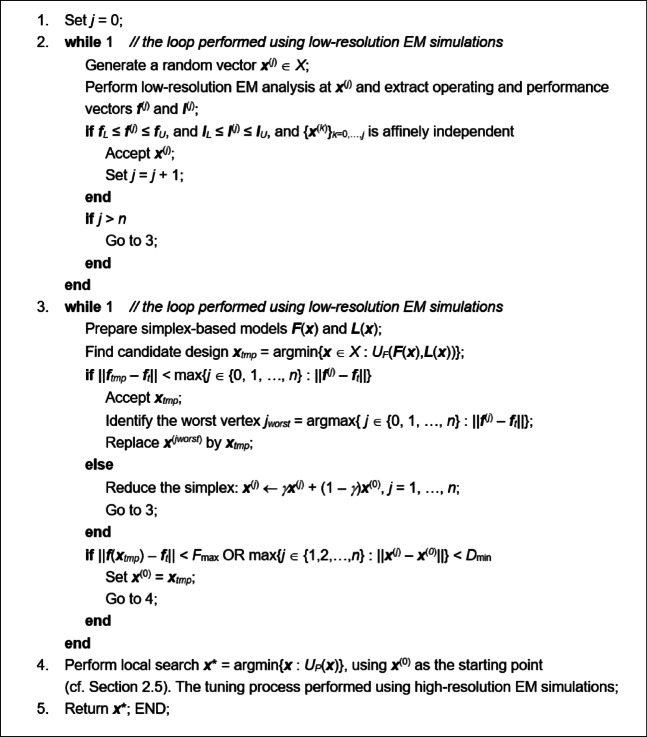
Proposed optimization procedure: pseudocode.

The control parameters are summarized in Table 1. One needs to emphasize that none of these is critical for the algorithm performance, except *F*_max_ (cf. Sect. Simplex refinement for more details). Most parameters control the resolution of the optimization process. Figures 7 and 8 provide the flow diagram and the pseudocode of the suggested procedure.

**Table 1:** Proposed optimization algorithm: control parameters.

Parameter	Explanation	Value
*F*_max_	Threshold for terminating the global search stage (cf. (16))	Problem-dependent, typically, a fraction of GHz
*α*	Search region extension parameter (cf. (12))	0.2
*γ*	Simplex reduction ratio (cf. (15))	0.5
*D*_min_	Termination parameter (global search)	1% of the parameter space size
*ε*	Termination parameter (local tuning)	10^–3^
*M*_*c*_	Multiplication factor for enabling sensitivity update using Broyden formula	10

## Algorithm verification and benchmarking

Our algorithm is comprehensively validated and juxtaposed with benchmark procedures that include bio-inspired techniques, randomized gradient search, and a simplex-based routine using high-fidelity EM simulations. The verification set comprises four microstrip antennas. The primary performance indicators are global search capability, design quality, and computational efficiency.

### Verification antennas

**Fig. 9:**
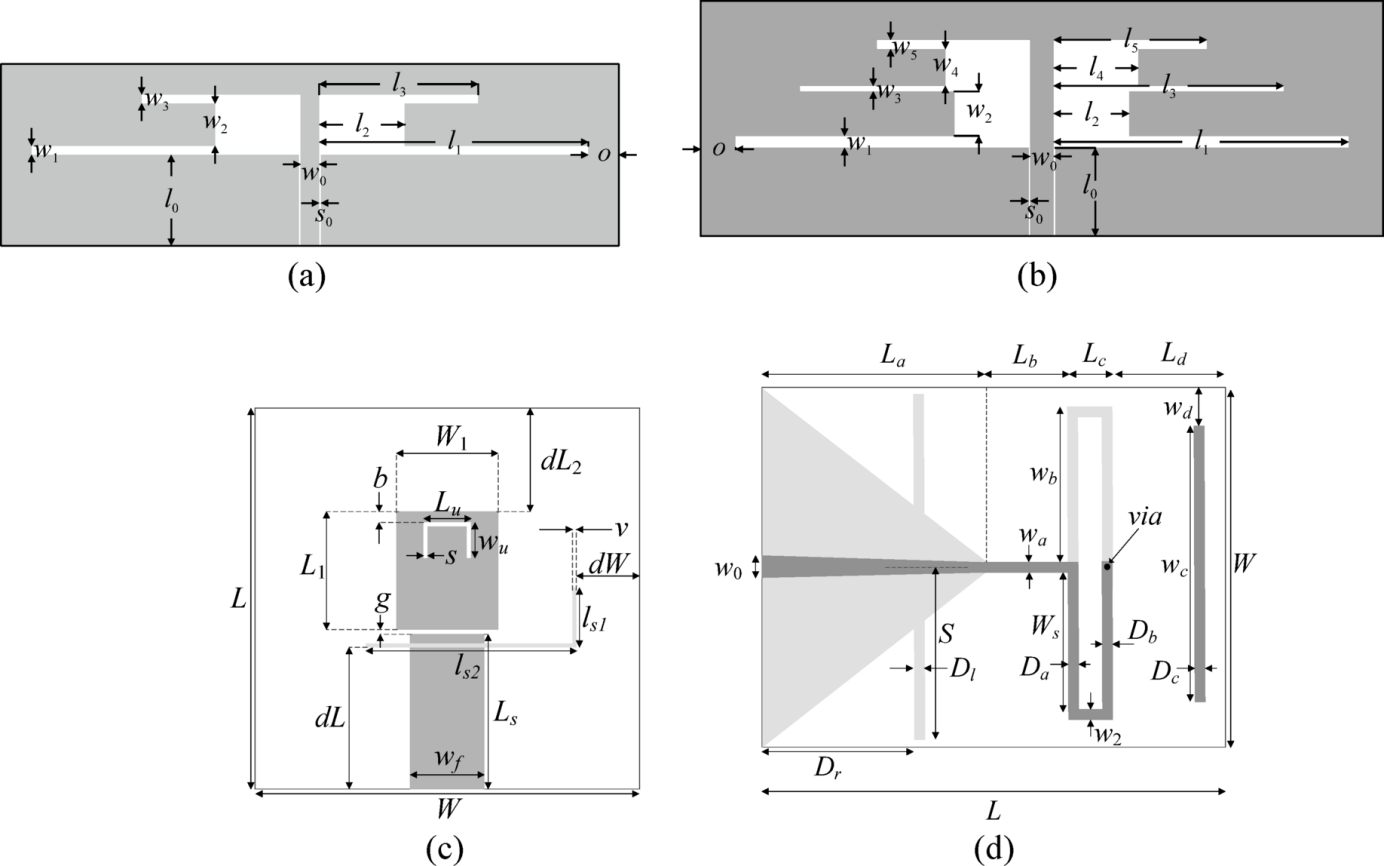
Verification test cases: (**a**) Antenna I (dual-band dipole)^110^, (**b**) Antenna II (triple-band dipole)^111^, (**c**) Antenna III (triple-band patch with defected ground; ground slot shown using light gray)^112^, (**d**) Antenna IV (quasi-Yagi with integrated balun, shown using light gray)^113^.

**Fig. 10:**
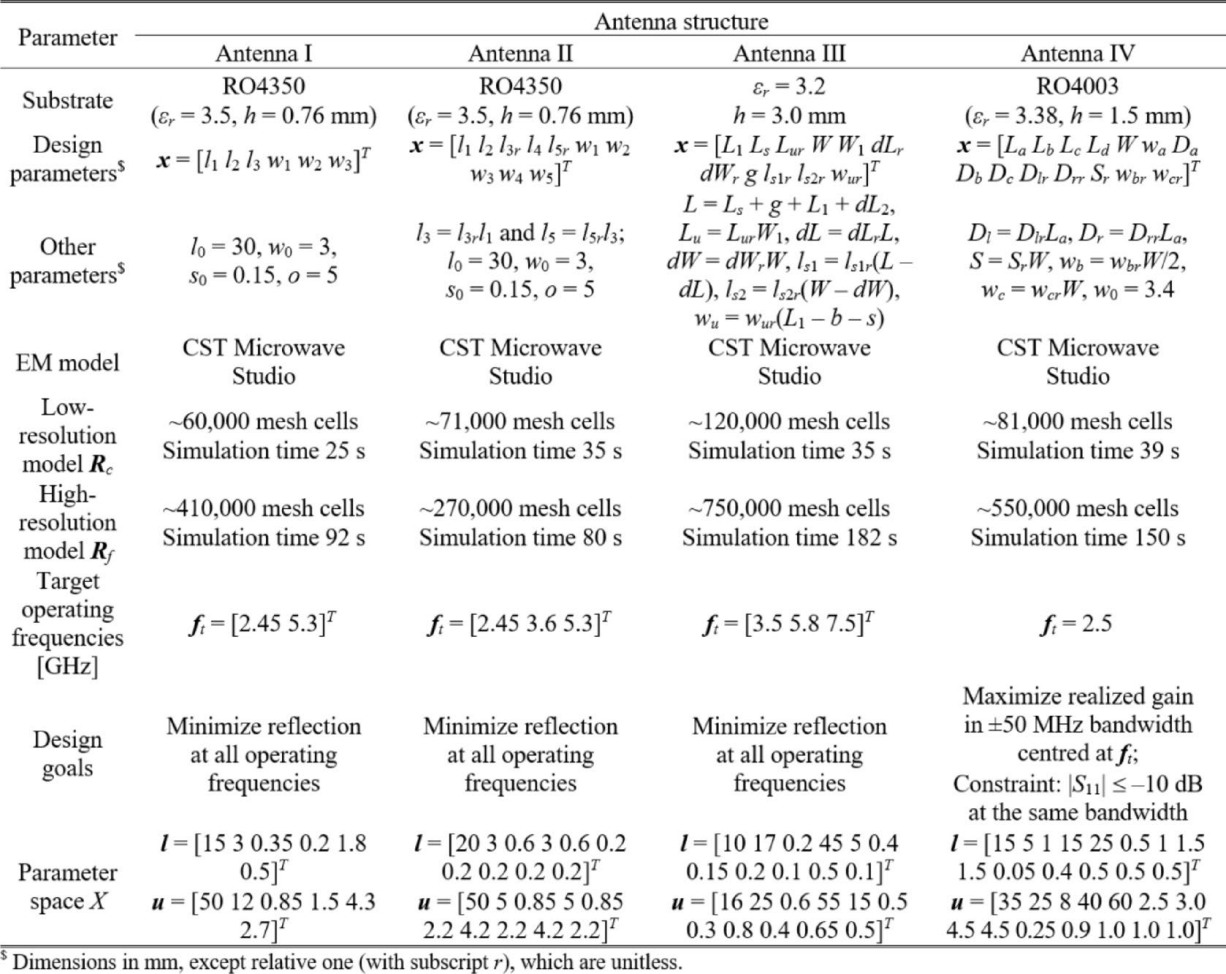
Important parameters of antennas of Fig. 9.

Consider microstrip antennas shown in Fig. 9^110–113^. Figure 10 provides essential data concerning substrate materials, decision variables, EM models, and the target operating frequency vectors ***f***_*t*_. For Antennas I through III, the performance vector ***l*** (cf. Figure 7) contains the antenna reflection coefficient |*S*_11_| at the resonances; for Antenna IV, it also includes the maximum in-band gain.

The EM models ***R***_*c*_ and ***R***_*f*_ are evaluated in CST Microwave Studio. The analysis resolution is adjusted using the lines-per-wavelength parameter. ***R***_*c*_ is set up to ensure that the EM analysis yields all important response features (e.g., resonances), whereas ***R***_*f*_ is adjusted based on the grid convergence. As observed in Fig. 10, ***R***_*c*_ is 2.3 (for Antenna II) to 5.2 (Antenna III) faster than ***R***_*f*_. Clearly, the computational savings being a result of incorporating variable resolution models depends on that ratio.

Antennas I, II, and III are optimized for best impedance matching. The associate objective function is defined as *U*(***x***) = max{*k* = 1, …, *K* : |*S*_11_(***x***,*f*_0.*k*_)|}, where *K* is the number of frequency bands (two for Antenna I, and three for Antennas II and III), whereas *f*_0.*k*_ is the *k*th center frequency, cf. target operating frequencies in Fig. 10). Antenna IV is optimized for maximum gain *G*(***x***,*f*_0_) and the impedance matching condition |*S*_11_(***x***)| ≤ − 10 dB over the ± 50 MHz frequency range *F* centered at *f*_0_ = 2.5 GHz. The corresponding objective function is defined as *U*(***x***) = –*G*(***x***,*f*_0_) + *βc*(***x***)^2^, where the penalty coefficient *β* = 100, and the penalty function is *c*(***x***) = max{ (max{*f* ∈ *F* : |*S*_11_(***x***,*f*)|} + 10)/10, 0}. The penalty term enforces the impedance matching condition, whereas maximization of gain is the primary goal.

### Numerical experiment setup

The antennas of Fig. 9 were optimized using our algorithm and several benchmark procedures outlined in Table 2. Our framework was run with the default setup, i.e., *F*_max_ = 0.2 GHz, *α* = 0.2, *γ* = 0.5, *D*_min_ = 1, *ε* = 10^–3^ and *M*_*c*_ = 10 (cf. Table 2). This allows us to demonstrate that no problem-related algorithm tuning is necessary.

**Table 2:** Algorithm setup (proposed and benchmark).

Algorithm	Name	Outline
This study	Variable-resolution simplex-based global search with gradient-based fine tuning (Sect. Global optimization with simplex regressors and variable-resolution models)	Control parameters: *F*_max_ = 0.2 GHz, *α* = 0.2, *γ* = 0.5, *D*_min_ = 1, *ε* = 10^–3^ and *M*_*c*_ = 10 (cf. Table 1)
I	Particle swarm optimizer (PSO)	Swarm size *N* = 10, number of iterations: 50 (Version I) and 100 (Version II); conventional parameter setup (*χ* = 0.73, *c*_1_ = *c*_2_ = 2.05);
II	Differential evolution (DE)	Population size *N* = 10, number of iterations set to 50 (Version I) and 100 (Version II); conventional parameter setup (*CR* = 0.5, *F* = 1)^114^;
III	Trust-region (TR) gradient-based optimizer [107]	Random initialization, Jacobian matrix estimated by finite differentiation, termination: convergence in argument [115]
IV	Machine-learning procedure	Algorithm setup: Initial surrogate set up to ensure relative RMS error less than 20% (max. number of training samples limited to 400); the algorithm operates in the original parameter space (no dimensionality reduction); infill criterion: minimization of the predicted objective function; surrogate model optimization using the particle swarm optimizer
V	Simplex-based global search with gradient-based fine tuning	Algorithm setup: the same as for the proposed approach, except that the search process is conducted using ***R***_*f*_

The benchmark methods, PSO, DE, gradient search, a machine learning algorithm employing kriging interpolation surrogates, and simplex-based global search using high-resolution EM analysis, have been chosen to verify the relevance of the specific features of the proposed approach. For example, a comparison with PSO and DE allows us to illustrate computational advantages of our framework over nature-inspired routines. Note that both were set up with a low budget for population-based procedures (only 500 and 1000 function calls for Version I and II, respectively). These budgets, although low from nature-inspired optimization perspective, are considerable for practical EM-based design (two to three days of CPU time per algorithm run on average). The inclusion of gradient-based search (Algorithm III) allows us to corroborate multimodality of the considered test problems. The machine learning algorithm is incorporated to illustrate the operation of a surrogate-assisted approach; here, based on the iterative prediction-correction scheme, where the surrogate model yields predictions concerning the location of the optimum design, and it is refined using the accumulated EM simulation data. Finally, a comparison with Algorithm IV, which essentially coincides with the proposed one except of being executed exclusively using ***R***_*f*_, enables demonstrating the computational benefits of the capitalizing of multi-resolution models, and investigating whether the employment of ***R***_*f*_ early in the search process compromises dependability.

### Findings

**Fig. 11:**
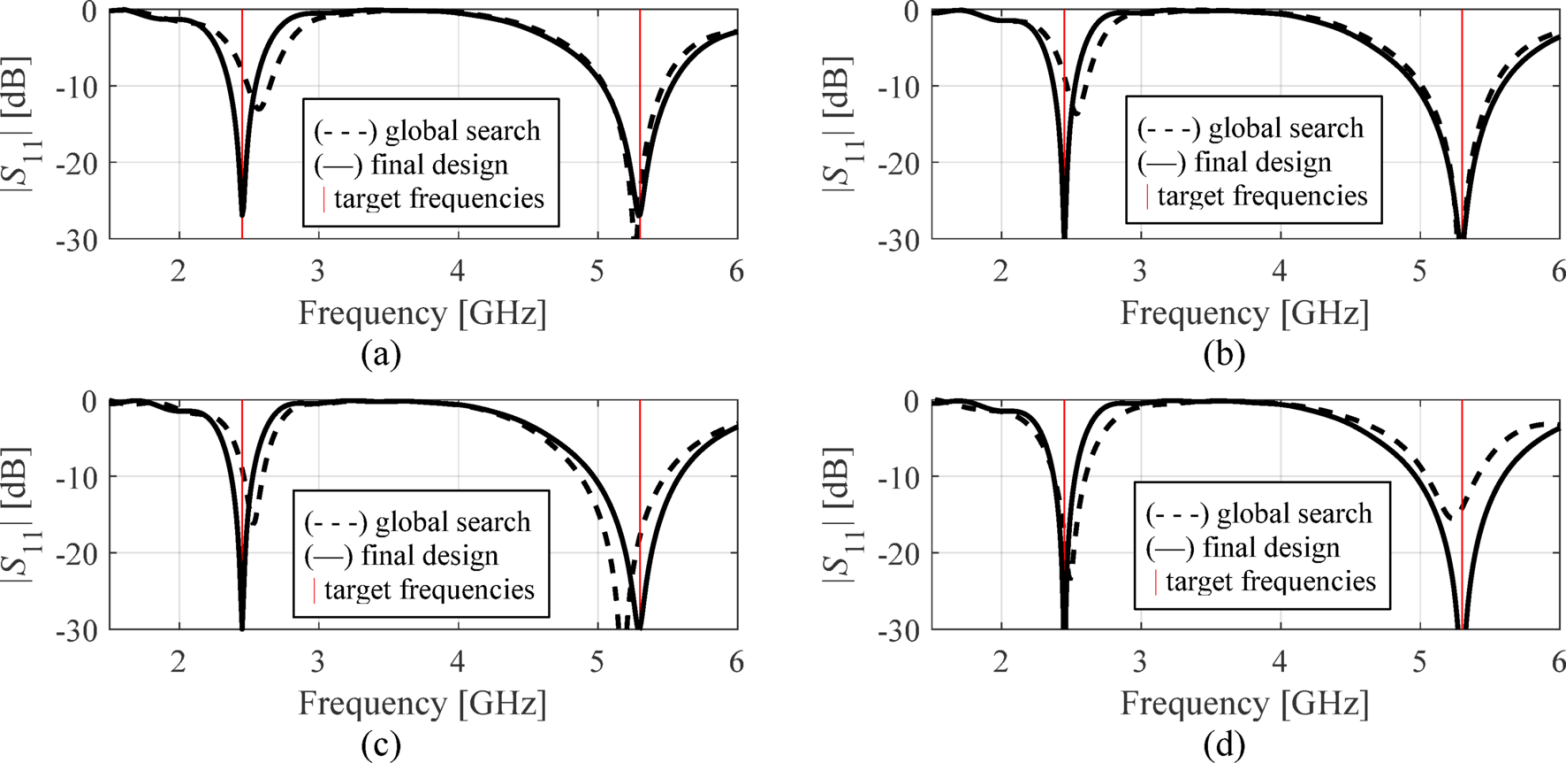
Antenna I: frequency characteristics at the designs found using our algorithm for representative runs: (**a**) run 1, (**b**) run 2, (**c**) run 3, (**d**) run 4.

**Fig. 12:**
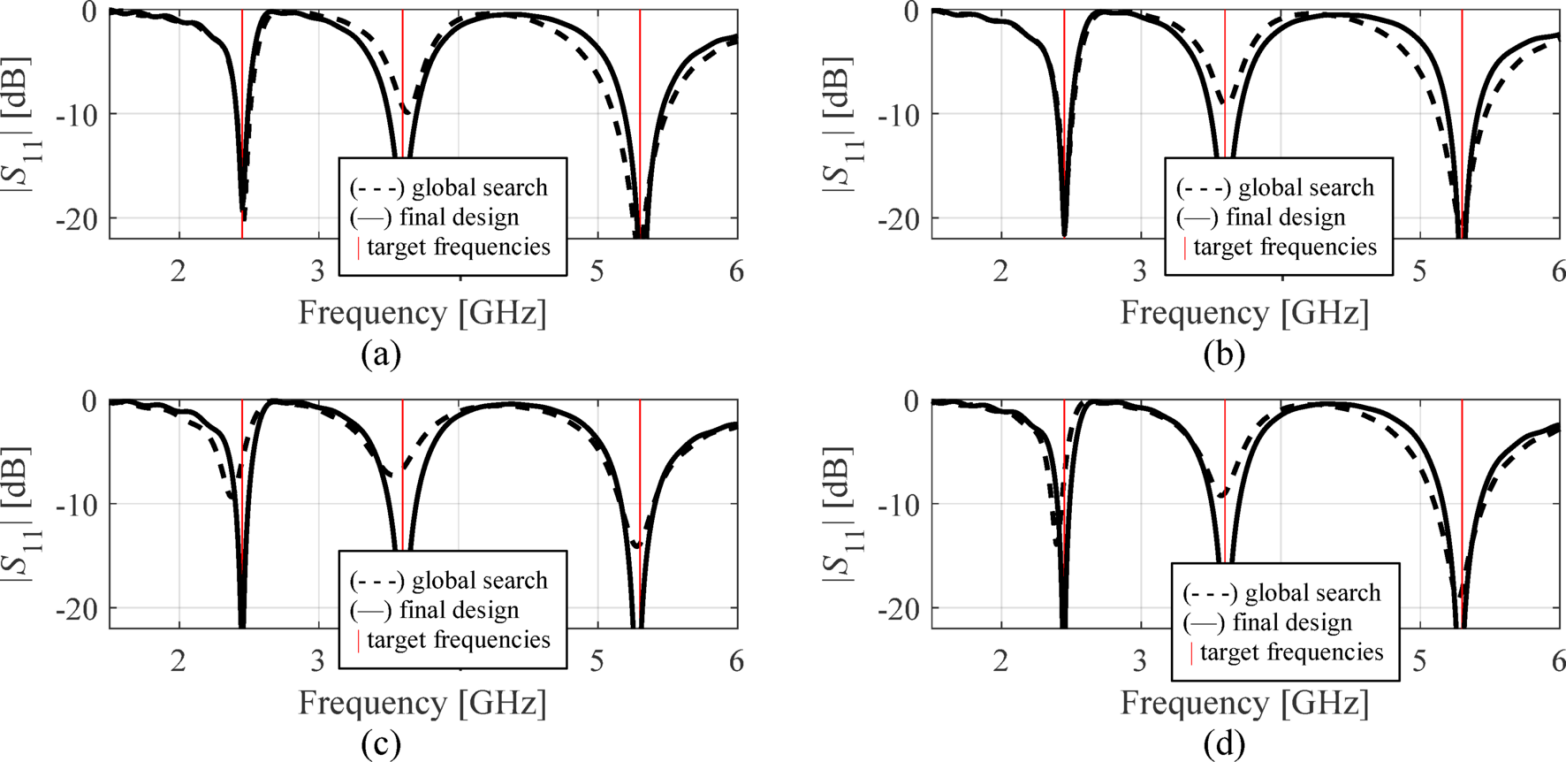
Antenna II: frequency characteristics at the designs found using our algorithm for representative runs: (**a**) run 1, (**b**) run 2, (**c**) run 3, (**d**) run 4.

**Fig. 13:**
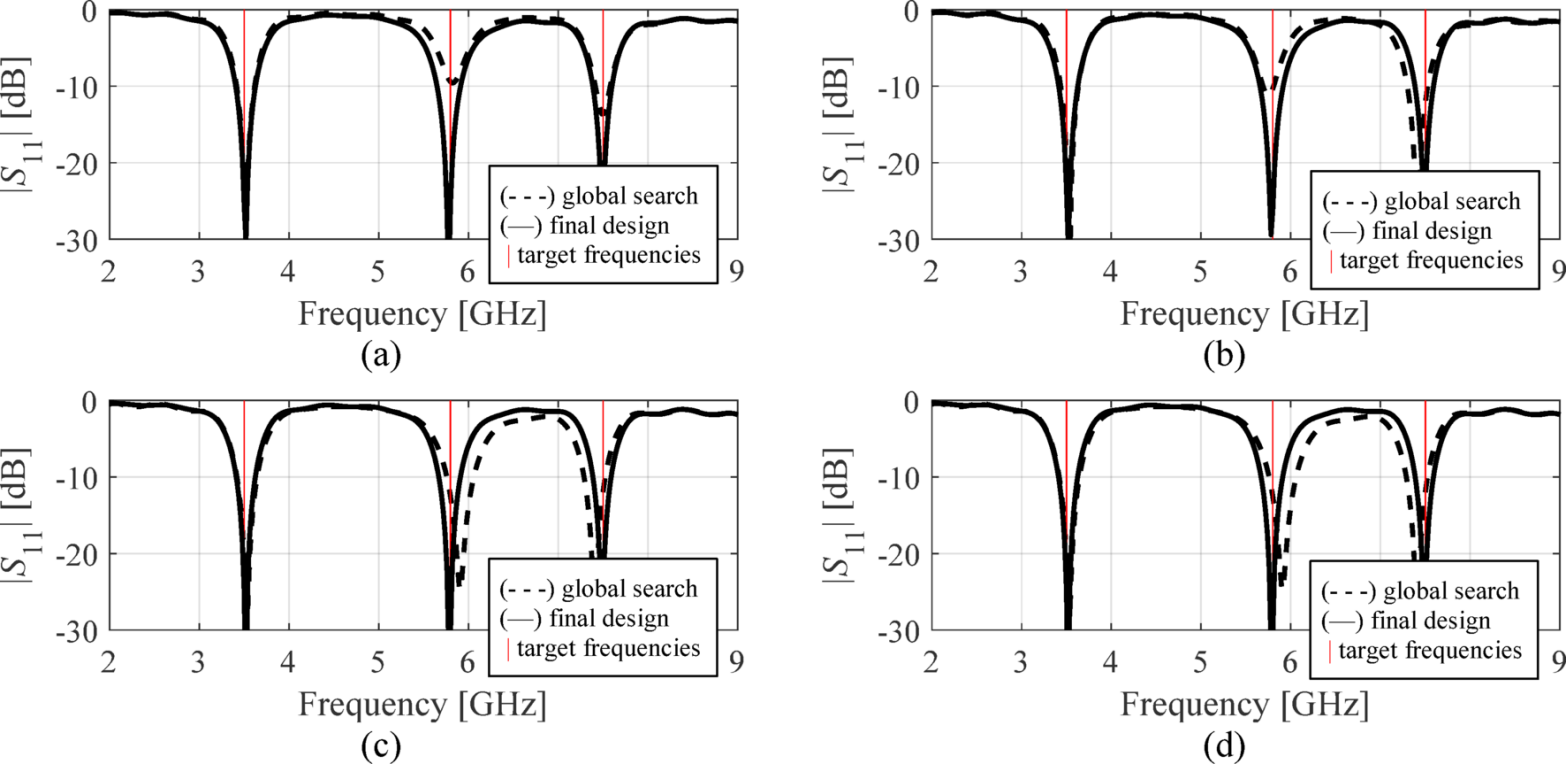
Antenna III: frequency characteristics at the designs found using our algorithm for representative runs: (**a**) run 1, (**b**) run 2, (**c**) run 3, (**d**) run 4.

**Fig. 14:**
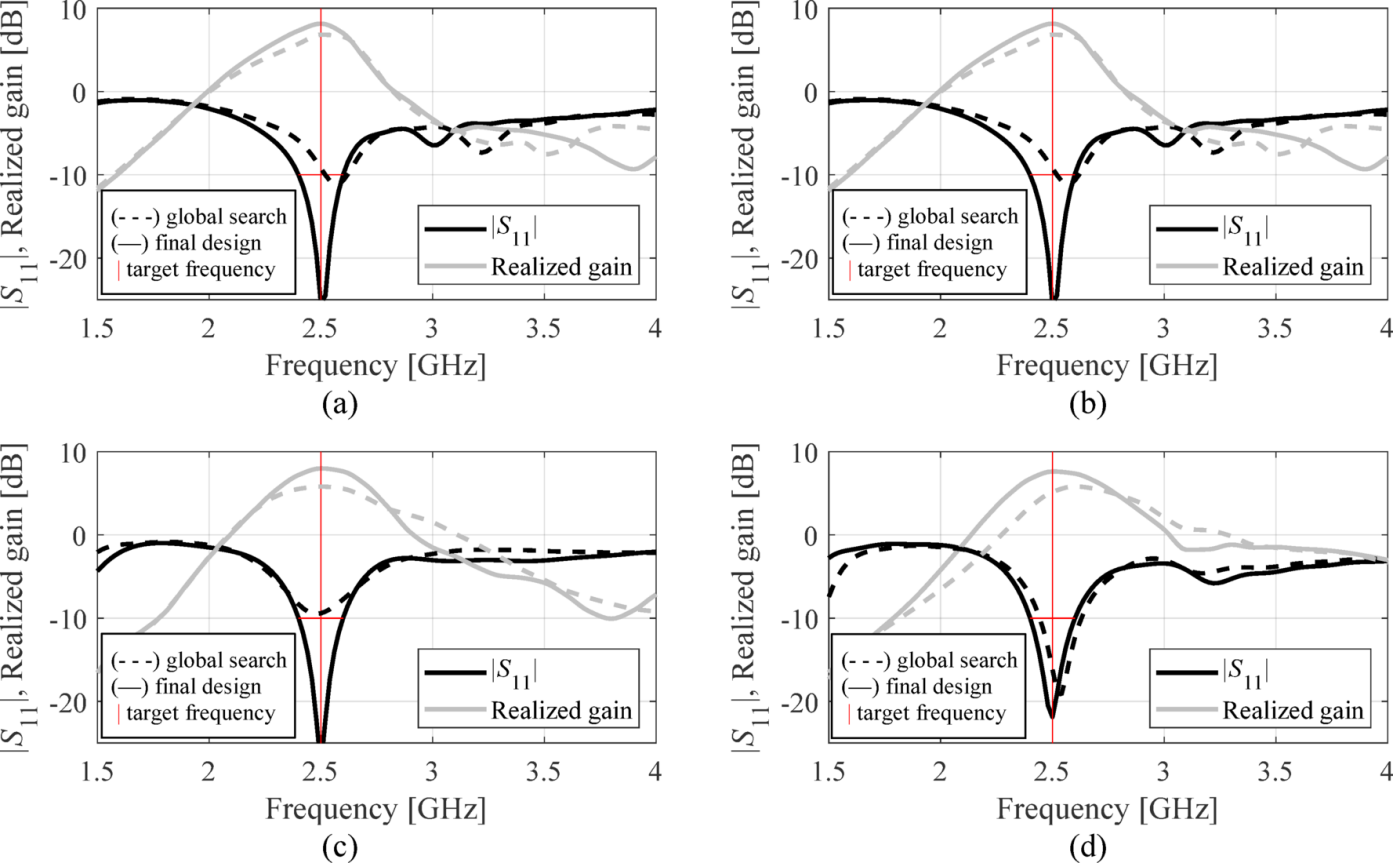
Antenna IV: frequency characteristics at the designs found using our algorithm for representative runs: (**a**) run 1, (**b**) run 2, (**c**) run 3, (**d**) run 4.

The results are gathered in Tables 3, 4 and 5, and 6 for Antennas I through IV. Figures 11, 12 and 13, and 14 illustrate antenna outputs at the designs produced by the global stage and the final outcomes (obtained through local tuning) for selected algorithm runs. The numbers in square brackets refer to the total optimization time that includes the cost of EM simulations and other components (e.g., surrogate model optimization time in the case of Algorithm IV, etc.).

**Table 3:** Results for antenna I.

Method	This work	PSO (Algorithm I)		DE (Algorithm II)		TR algorithm (Algorithm III)	Machine learning with kriging surrogates (Algorithm IV)	Simplex-based procedure using high-resolution EM model (Algorithm V)
		50 iterations	100 iterations	50 iterations	100 iterations			
Mean cost function [dB]	–30.9	–18.2	–19.3	–21.5	–22.8	–13.5	–20.7	–25.3
Computational cost^$^	65.3 [1.7 h]	500 [12.8 h]	1,000 [25.6 h]	500 [12.8 h]	1,000 [25.6 h]	84.2 [2.2 h]	457.8 [11.9 h]	82.9 [2.1 h]
Success rate^#^	10/10	9/10	10/10	9/10	9/10	6/10	10/10	10/10

^$^Equivalent number of high-resolution model evaluations. The numbers in brackets refer to the total optimization time in hours.

^#^Number runs for which ||***f***(x^*^) – ***f***_*t*_|| < *F*_max_.

**Table 4:** Results for antenna II.

Method	This work	PSO (Algorithm I)		DE (Algorithm II)		TR algorithm (Algorithm III)	Machine learning with kriging surrogates (Algorithm IV)	Simplex-based procedure using high-resolution EM model (Algorithm V)
		50 iterations	100 iterations	50 iterations	50 iterations			
Mean cost function [dB]	–20.1	–10.8	–13.8	–12.1	–13.6	–7.8	–13.5	–17.5
Computational cost^$^	122.0 [2.7 h]	500 [11.1 h]	1,000 [22.3 h]	500 [11.1 h]	1,000 [22.3 h]	105.8 [2.4 h]	470.0 [10.7 h]	154.0 [3.4 h]
Success rate^#^	10/10	5/10	8/10	6/10	8/10	4/10	10/10	10/10

^$^Equivalent number of high-resolution model evaluations. The numbers in brackets refer to the total optimization time in hours.

^#^Number runs for which ||***f***(x^*^) – ***f***_*t*_|| < *F*_max_.

**Table 5:** Results for antenna III.

Method	This work	PSO (Algorithm I)		DE (Algorithm II)		TR algorithm (Algorithm III)	Machine learning with kriging surrogates (Algorithm IV)	Simplex-based procedure using high-resolution EM model (Algorithm V)
		50 iterations	100 iterations	50 iterations	100 iterations			
Mean cost function [dB]	–20.7	–12.3	–14.2	–13.5	–15.1	–12.1	–11.8	–17.5
Computational cost^$^	81.1 [4.1 h]	500 [25.3 h]	1,000 [50.6 h]	500 [25.3 h]	1,000 [50.6 h]	125.4 [25.3 h]	471.6 [24.1 h]	110.7 [5.6 h]
Success rate^#^	10/10	6/10	8/10	7/10	9/10	4/10	9/10	10/10

^$^Equivalent number of high-resolution model evaluations. The numbers in brackets refer to the total optimization time in hours.

^#^Number runs for which ||***f***(x^*^) – ***f***_*t*_|| < *F*_max_.

**Table 6:** Results for antenna IV.

Method	This work	PSO (Algorithm I)		DE (Algorithm II)		TR algorithm (Algorithm III)	Machine learning with kriging surrogates (Algorithm IV)	Simplex-based procedure using high-resolution EM model (Algorithm V)
		50 iterations	100 iterations	50 iterations	100 iterations			
Mean cost function [dB]^&^	7.6	6.1	6.8	6.0	6.9	–1.1	7.9	7.4
Computational cost^$^	115.1 [4.8 h]	500 [20.8 h]	1,000 [41.7 h]	500 [20.8 h]	1,000 [41.7 h]	138.4 [5.8 h]	583.3 [24.5 h]	144.3 [6.0 h]
Success rate^#^	10/10	9/10	10/10	8/10	10/10	1/10	10/10	10/10

^&^Realized gain at 2.5 GHz.

^$^Equivalent number of high-resolution model evaluations. The numbers in brackets refer to the total optimization time in hours.

^#^Number runs for which ||***f***(x^*^) – ***f***_*t*_|| < *F*_max_.

Each technique has been executed ten times for all antenna structures, and the mean cost function values along with the running expenses are reported in the tables. An additional performance indicator is the success rate, i.e., the fraction of runs where ||***f***(x^*^) – ***f***_*t*_|| < *F*_max_, i.e., the actual operating parameters are sufficiently close to the target. The results collected in Tables 3, 4, 5, and 6 are analyzed below, and the proposed approach is compared to the benchmark methods with respect to computational efficiency, reliability, and design quality.

Reliability of the optimization process can be assessed using the success rate, which is perfect (i.e., 10/10) for the proposed framework across the entire antenna test set. This can also be viewed as confirmation of the procedure’s global search capabilities. Meanwhile, a low success rate (the average of 4/10) for TR-based search (Algorithm III) confirms design task multimodality. Among the benchmark methods, Algorithms IV and V also demonstrate 10/10 success, which is expected for Algorithm V because it shares the same operating principles with the proposed framework. Algorithm IV is slightly worse (the success rate for Antenna III is 9/10), yet it is generally comparable to the proposed approach. The performance of bio-inspired routines is noticeably inferior: the average success rate is about 7/10 for Version I (500 objective function calls), and about 9/10 for Version II (1,000 objective function calls) for both PSO and DE. This difference is indicative of insufficient computational budget assigned to these methods (set to avoid excessive CPU costs as explained earlier). It is likely that increasing the budget to, say, 2,000 function calls, would improve PSO/DE performance even further.

In terms of design quality, the proposed method and Algorithm IV are superior to other techniques, which demonstrates the relevance of the algorithmic tools employed here, in particular, conducting the global search stage using the operating figures. At the same time, the results demonstrate that the speedup achieved by employing multi-resolution EM analysis does not jeopardize dependability. Quite the opposite: the mean cost function is slightly better for the proposed algorithm than for Algorithm III; however, the differences are not significant. In detail, regarding the average value of the objective function, the proposed algorithm provides considerably better results than all benchmark techniques. For Antennas I, II, and III, the cost function is expressed in a minimax form and refers to the maximum in-band reflection level, which should be as low as possible (and lower than − 10 dB). As shown in Tables 3 and 4, and 5, our technique yields objective function values better than the benchmark by at least several decibels. Only the single-fidelity simplex-based procedure renders comparable results yet still noticeably worse results. For Antenna IV, the goal was the enhancement of the end-fire realized gain. In this case, the average results provided by our method are considerably better than all benchmark methods except Algorithms IV and V. The former yields slightly better results (by 0.3 dB on average), whereas the results of the latter are marginally worse (by 0.2 dB on average).

The efficiency of our algorithm is remarkable. The typical cost corresponds to just 95 high-resolution EM analyzes, which is 23% less than for Algorithm IV. This difference demonstrates the role of employing ***R***_*c*_ early in the search process. Interestingly, the cost is lower than for local tuning (Algorithm III). At first glance, this result seems surprising, because the proposed framework contains local tuning as one of its components. However, the final parameter tuning within our algorithm starts from a very good initial design produced by the simplex-based search at the low-resolution level, which greatly lowers the overall expenses. Comparison with PSO and DE (Version II with the budgets of 1,000 function calls) reveals dramatic speedup: the savings exceed 90% and would be significantly higher should bio-inspired algorithms be run under more realistic budgets. Finally, our technique is substantially faster than the machine learning routine (Algorithm IV): the expenses incurred by the latter are around 500 EM simulations on average, which is around five times more than for the proposed algorithm. Also, except for Antenna IV, Algorithm IV provides much worse values of the objective function. The main reason is the necessity building a reasonable surrogate model over the parameter space, which is impeded by its large size but also dimensionality-related problems.

The performance of the proposed framework makes it suitable for solving global optimization problems under challenging scenarios. Because the search process is conducted using operating parameters, the method is predisposed to handle structures for which identification of the operating figures is straightforward (in particular, multi-band antennas). Excellent computational efficiency and no need of tuning the procedure for a specific problem are additional advantages that make our framework an attractive alternative to conventional global optimization techniques such as nature-inspired routines.

It should be emphasized that the proposed algorithm is a global search procedure. As such, it does not require any initial design. The optimization process is conducted in the entire parameter space. However, due to the nature of the algorithm, there are some potential limitations of the method. In particular, setting up the simplex-based regression models is contingent upon the extraction of the operating frequencies of the antenna, which is based on random sampling (Sect. Simplex-Based regression models, Fig. 8) and may be problematic for poor-quality designs (due to heavily distorted responses). This would lead to the rejection of most of the random observables at the sampling stage, consequently increasing the optimization cost. The problem would be pronounced for very large search spaces, which are arbitrarily set up without accounting for the engineering insight that might help establish reasonable ranges of design variables. On the other hand, a reasonable establishment of the parameter space allows for effective mitigation of this issue. In fact, the parameter spaces assumed for Antennas I through IV considered in our verification studies are large regarding dimensionality and parameter ranges; still, our technique proved to be successful.

### Experimental validation

**Fig. 15:**
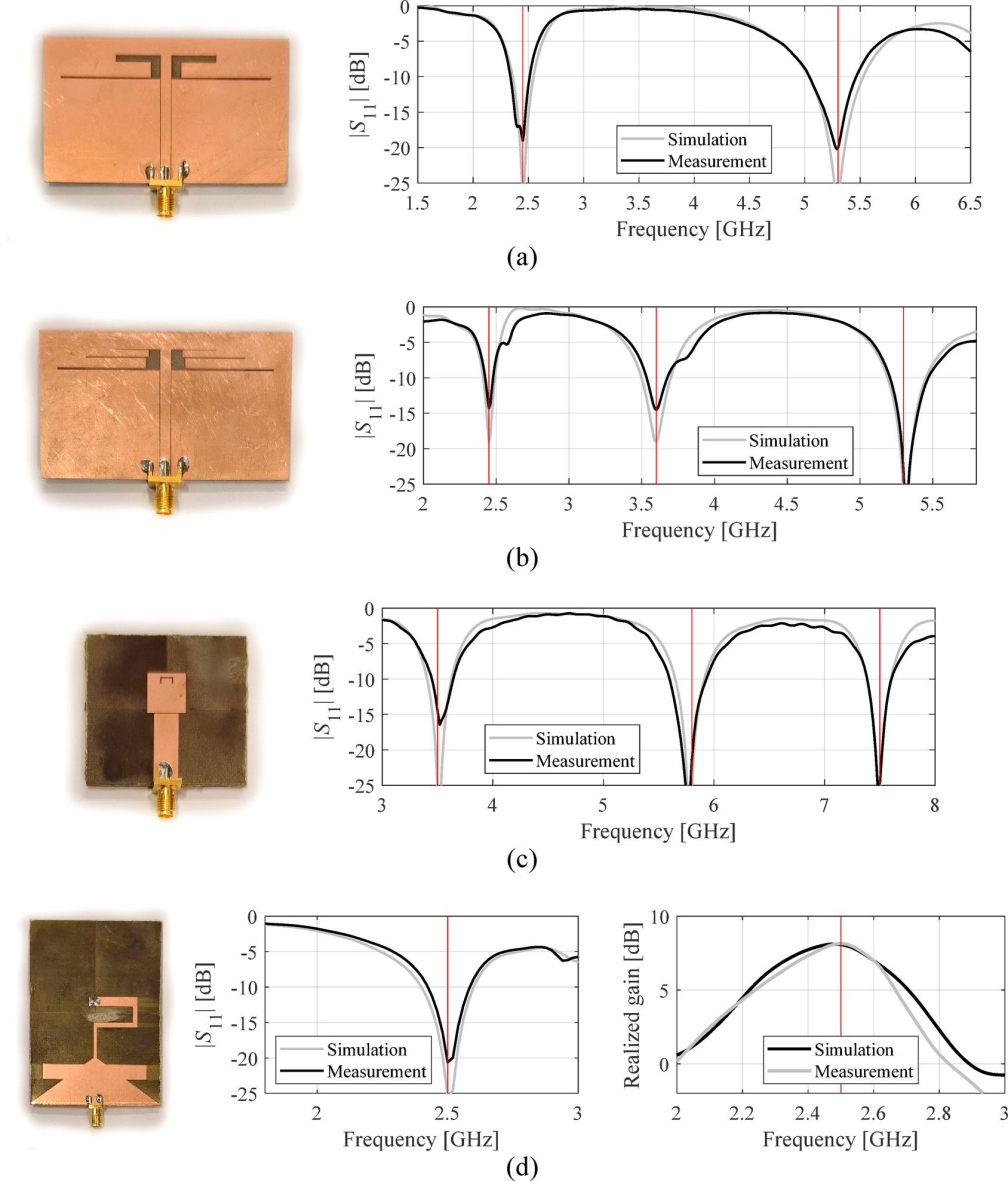
Prototypes of Antennas I through IV and comparison of EM analysis (gray) and measurements (black): (**a**) Antenna I, (**b**) Antenna II, (**c**) Antenna III, (**d**) Antenna IV.

**Fig. 16:**
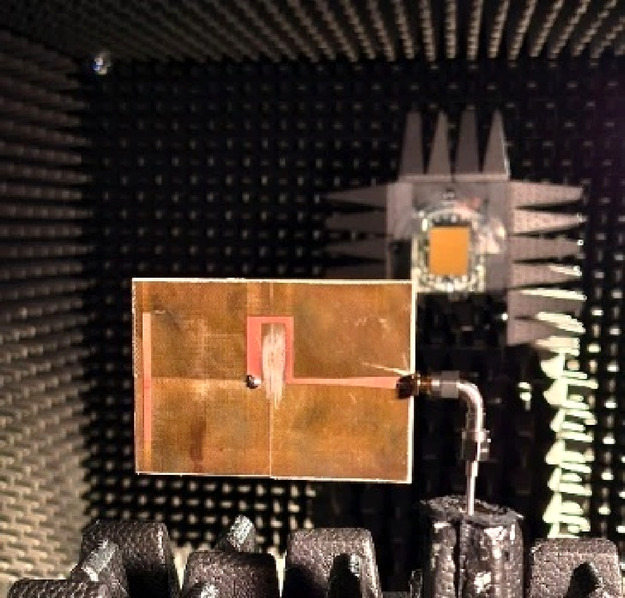
Measurement setup (here, shown for Antenna IV).

The designs optimized using our algorithm were manufactured and experimentally validated to provide a supplementary illustration. The specific designs considered here are those included in Figs. 11(a) (Antenna I), 12(a) (Antenna II), 13(a) (Antenna III), and 14(a) (Antenna IV). Figure 15 shows the prototypes and a comparison between EM analysis and measurements. In the case of Antenna IV, the measured end-fire gain is also included as this parameter was subject to optimization for this structure. Figure 16 shows the measurement setup for Antenna IV in the anechoic chamber. The setup includes a double-rigged horn antenna (Geozondas GZ0226DRH) and Anritsu VNA (MS4644B). The alignment between EM simulation and measurements is satisfactory. Minor discrepancies can be attributed to manufacturing inaccuracies.

## Conclusion

In this research, we introduced an innovative methodology for global antenna optimization. Our technique employs variable-resolution EM models, simplex-based surrogates, and gradient-based final tuning. The presented framework underwent numerical verification involving four antennas and several benchmark procedures. The results demonstrate the ability of the proposed technique to allocate operating frequencies close to the targets in all algorithm runs. The quality of results, measured by the achieved objective function value, is competitive with the benchmark. Computational efficiency is remarkable, with the average running costs corresponding to only 95 EM simulations of the antenna at the high-resolution level. These costs represent over 23% savings over the simplex-based procedure using high-resolution EM simulations and 91% savings regarding the nature-inspired optimization. Another advantage of our approach is simple handling owing to a limited number of control parameters and no need for problem-specific algorithm tuning.

Conducting the search process using the operating parameters constitutes a potential limitation of the proposed technique. In particular, setting up the simplex-based regression models requires extraction of the operating frequencies, which may be problematic for poor-quality designs (due to heavily distorted responses). This would lead to rejecting the majority of random observables at the sampling stage, consequently increasing the optimization cost. Nonetheless, a reasonable establishment of the parameter space through engineering insight allows for effective mitigation of this issue. Given the aforementioned reservation, the suggested framework might be considered an attractive and cost-efficient alternative to available optimization methods, including bio-inspired and machine-learning algorithms.

## Data Availability

The datasets used and/or analyzed during the current study are available from the corresponding author on reasonable request.

## References

[1] Zhang, Y., Deng, J., Li, M., Sun, D. & Guo, L. „A MIMO dielectric resonator antenna with improved isolation for 5G mm-wave applications. *IEEE Ant Wirel. Propag. Lett.* **18** (4), 747–751 (2019).

[2] Wen, S. & Dong, Y. „A low-profile wideband antenna with monopolelike radiation characteristics for 4G/5G indoor micro base station application. *IEEE Ant Wirel. Propag. Lett.* **19** (12), 2305–2309 (2020).

[3] Jha, K. R., Bukhari, B., Singh, C., Mishra, G. & Sharma, S. K. „Compact planar multistandard MIMO antenna for IoT applications. *IEEE Trans. Ant Propag.* **66** (7), 3327–3336 (2018).

[4] Moon, S. M., Yun, S., Yom, I. B. & Lee, H. L. Phased array shaped-beam satellite antenna with boosted-beam control. *IEEE Trans. Ant Propag.* **67** (12), 7633–7636 (2019).

[5] Mansour, M. M. & Kanaya, H. High-efficient broadband CPW RF rectifier for wireless energy harvesting. *IEEE Microw. Wirel. Comp. Lett.* **29** (4), 288–290 (2019).

[6] Lin, X. et al. „Ultrawideband textile antenna for wearable microwave medical imaging applications. *IEEE Trans. Ant Propag.* **68** (6), 4238–4249 (2020).

[7] Kapusuz, K. Y., Berghe, A. V., Lemey, S. & Rogier, H. Partially filled half-mode substrate integrated waveguide leaky-wave antenna for 24 ghz automotive radar. *IEEE Ant Wirel. Propag. Lett.* **20** (1), 33–37 (2021).

[8] Ullah, U., Koziel, S. & Mabrouk, I. B. Rapid re-design and bandwidth/size trade-offs for compact wideband circular polarization antennas using inverse surrogates and fast EM-based parameter tuning. *IEEE Trans. Ant Prop.* **68** (1), 81–89 (2019).

[9] He, Y., Yue, Y., Zhang, L. & Chen, Z. N. A dual-broadband dual-polarized directional antenna for all-spectrum access base station applications. *IEEE Trans. Ant Propag.* **69** (4), 1874–1884 (2021).

[10] Sun, L., Li, Y., Zhang, Z. & Feng, Z. „Wideband 5G MIMO antenna with integrated orthogonal-mode dual-antenna pairs for metal-rimmed smartphones. *IEEE Trans. Ant Propag.* **68** (4), 2494–2503 (2020).

[11] Hynes, C. G. & Vaughan, R. G. „Conical monopole antenna with integrated tunable Notch filters. *IEEE Ant Wirel. Propag. Lett.* **19** (12), 2398–2402 (2020).

[12] Liu, J., Tang, Z., Wang, Z., Li, H. & Yin, Y. „Gain enhancement of a broadband symmetrical dual-loop antenna using shorting pins. *IEEE Ant Wirel. Propag. Lett.* **17** (8), 1369–1372 (2018).

[13] Rabbani, M. S., Churm, J. & Feresidis, A. P. „Continuous beam-steering low-loss millimeter-wave antenna based on a piezo-electrically actuated metasurface. *IEEE Trans. Ant Propag.* **70** (4), 2439–2449 (2022).

[14] Yu, H., Yu, J., Yao, Y., Liu, X. & Chen, X. Wideband circularly polarized Horn antenna exploiting open slotted end structure. *IEEE Ant Wirel. Propag. Lett.* **19** (2), 267–271 (2020).

[15] Hu, W., Yin, Y., Yang, X. & Fei, P. „Compact multiresonator-loaded planar antenna for multiband operation. *IEEE Trans. Ant Propag.* **61** (5), 2838–2841 (2013).

[16] Podilchak, S. K., Johnstone, J. C., Caillet, M., Clénet, M. & Antar, Y. M. M. „A compact wideband dielectric resonator antenna with a meandered slot ring and cavity backing. *IEEE Ant Wirel. Propag. Lett.* **15**, 909–913 (2016).

[17] Haq, M. A., Koziel, S. & Cheng, Q. S. Miniaturization of wideband antennas by means of feed line topology alterations. *IET Microwaves Ant Prop.* **12** (13), 2128–2134 (2018).

[18] Zhang, Y. X., Jiao, Y. C. & Zhang, L. „Antenna array directivity maximization with sidelobe level constraints using convex optimization. *IEEE Trans. Ant Propag.* **69** (4), 2041–2052 (2021).

[19] Zhang, Z., Chen, H. C. & Cheng, Q. S. „Surrogate-assisted quasi-Newton enhanced global optimization of antennas based on a heuristic hypersphere sampling. *IEEE Trans. Ant Propag.* **69** (5), 2993–2998 (2021).

[20] Koziel, S. & Pietrenko, A. Rapid design centering of multi-band antennas using knowledge-based inverse models and response features, *Knowledge Based Systems*, vol. 252, paper no. 109360, (2022).

[21] Kovaleva, M., Bulger, D. & Esselle, K. P. Comparative study of optimization algorithms on the design of broadband antennas. *IEEE J. Multiscale Multiphysics Comp. Techn*. **5**, 89–98 (2020).

[22] Tansui, D. & Thammano, A. „Hybrid nature-inspired optimization algorithm: hydrozoan and sea turtle foraging algorithms for solving continuous optimization problems. *IEEE Access.* **8**, 65780–65800 (2020).

[23] Lei, S. et al. „Power gain optimization method for wide-beam array antenna via convex optimization. *IEEE Trans. Ant Prop.* **67** (3), 1620–1629 (2019).

[24] Easum, J. A., Nagar, J., Werner, P. L. & Werner, D. H. Efficient multiobjective antenna optimization with tolerance analysis through the use of surrogate models. *IEEE Trans. Ant Propag.* **66** (12), 6706–6715 (2018).

[25] Koziel, S. & Pietrenko-Dabrowska, A. Recent advances in accelerated multi-objective design of high-frequency structures using knowledge-based constrained modeling approach, *Knowledge Based Systems*, vol. 214, paper No. 106726, (2021).

[26] Li, Q., Chu, Q., Chang, Y. & Dong, J. Tri-objective compact log-periodic dipole array antenna design using MOEA/D-GPSO. *IEEE Trans. Ant Propag.* **68** (4), 2714–2723 (2020).

[27] Koziel, S. & Abdullah, M. Machine-learning-powered EM-based framework for efficient and reliable design of low scattering metasurfaces. *IEEE Trans. Microw. Theory Techn*. **69** (4), 2028–2041 (2021).

[28] Bayraktar, Z., Komurcu, M., Bossard, J. A. & Werner, D. H. The wind driven optimization technique and its application in electromagnetics. *IEEE Trans. Antennas Propag.* **61** (5), 2745–2757 (2013).

[29] Koziel, S. & Pietrenko-Dabrowska, A. Reliable EM-driven size reduction of antenna structures by means of adaptive penalty factors. *IEEE Trans. Ant Propag.* **70** (2), 1389–1401 (2021).

[30] Al-Azza, A. A., Al-Jodah, A. A. & Harackiewicz, F. J. Spider monkey optimization: a novel technique for antenna optimization. *IEEE Antennas Wirel. Propag. Lett.* **15**, 1016–1019 (2016).

[31] Shahid, R., Singh, A. K., Park, J. S., Park, S. O. & Koziel, S. Compact electromagnetic lens antennas using cascaded metasurfaces for gain enhancement and beam steering applications. *Int. J. RF Microw. CAE*10.1002/mmce.23327 (2022).

[32] Koziel, S. & Pietrenko-Dabrowska, A. Expedited acquisition of database designs for reduced-cost performance-driven modeling and rapid dimension scaling of antenna structures. *IEEE Trans. Ant Prop.* **69** (8), 4975–4987 (2021).

[33] Abdullah, M. & Koziel, S. A novel versatile decoupling structure and expedited inverse-model-based re-design procedure for compact single-and dual-band MIMO antennas. *IEEE Access.* **9**, 37656–37667 (2021).

[34] Yang, C., Zhang, J. & Tong, M. S. „An FFT-accelerated particle swarm optimization method for solving far-field inverse scattering problems. *IEEE Trans. Ant Propag.* **69** (2), 1078–1093 (2021).

[35] Liu, X., Du, B., Zhou, J. & Xie, L. „Optimal design of elliptical beam Cassegrain antenna. *IEEE Access.* **9**, 120765–120773 (2021).

[36] Kaur, S. et al. „Hybrid local-global optimum search using particle swarm gravitation search algorithm (HLGOS-PSGSA) for waveguide selection. *IEEE Access.* **9**, 127866–127882 (2021).

[37] Li, H. et al. „Newly emerging nature-inspired optimization - algorithm review, unified framework, evaluation, and behavioural parameter optimization. *IEEE Access.* **8**, 72620–72649 (2020).

[38] Goldberg, D. E. & Holland, J. H. *Genetic Algorithms and Machine Learning* (Springer, 1988).

[39] Michalewicz, Z. *Genetic algorithms + data Structures = evolution Programs* (Springer, 1996).

[40] Choi, K. et al. Hybrid algorithm combing genetic algorithm with evolution strategy for antenna design. *IEEE Trans. Magn.* **52**(3), 1–4 (2016).

[41] Zhu, D. Z., Werner, P. L. & Werner, D. H. Design and optimization of 3-D frequency-selective surfaces based on a multiobjective lazy ant colony optimization algorithm. *IEEE Trans. Ant Propag.* **65** (12), 7137–7149 (2017).

[42] Rayno, J., Iskander, M. F. & Kobayashi, M. H. „Hybrid genetic programming with accelerating genetic algorithm optimizer for 3-D metamaterial design. *IEEE Ant Wirel. Propag. Lett.* **15**, 1743–1746 (2016).

[43] Wang, D., Tan, D. & Liu, L. „Particle swarm optimization algorithm: an overview, *Soft Computing*, vol. 22, pp. 387–408, (2018).

[44] Jiang, Z. J., Zhao, S., Chen, Y. & Cui, T. J. „Beamforming optimization for time-modulated circular-aperture grid array with DE algorithm. *IEEE Ant Wirel. Propag. Lett.* **17** (12), 2434–2438 (2018).

[45] Baumgartner, P. et al. Multi-objective optimization of Yagi-Uda antenna applying enhanced firefly algorithm with adaptive cost function. *IEEE Trans. Magnetics*. **54** (3), 8000504 (2018).

[46] Li, X. & Luk, K. M. The grey Wolf optimizer and its applications in electromagnetics. *IEEE Trans. Ant Prop.* **68** (3), 2186–2197 (2020).

[47] Yang, S. H. & Kiang, J. F. Optimization of sparse linear arrays using harmony search algorithms. *IEEE Trans. Ant Prop.* **63** (11), 4732–4738 (2015).

[48] Prabhakar, S. K., Rajaguru, H. & Lee, S. „A framework for schizophrenia EEG signal classification with nature inspired optimization algorithms. *IEEE Access.* **8**, 39875–39897 (2020).

[49] Tang, W. J., Li, M. S., Wu, Q. H. & Saunders, J. R. „Bacterial foraging algorithm for optimal power flow in dynamic environments. *IEEE Trans. Circuits Syst. I: Regul. Papers*. **55** (8), 2433–2442 (2008).

[50] Zheng, T. et al. IWORMLF: improved invasive weed optimization with random mutation and lévy flight for beam pattern optimizations of linear and circular antenna arrays. *IEEE Access.* **8**, 19460–19478 (2020).

[51] Darvish, A. & Ebrahimzadeh, A. Improved fruit-fly optimization algorithm and its applications in antenna arrays synthesis. *IEEE Trans. Antennas Propag.* **66** (4), 1756–1766 (2018).

[52] Farghaly, S. I., Seleem, H. E., Abd-Elnaby, M. M. & Hussein, A. H. Pencil and shaped beam patterns synthesis using a hybrid GA/l₁ optimization and its application to improve spectral efficiency of massive MIMO systems. *IEEE Access.* **9**, 38202–38220 (2021).

[53] Liu, Z. Z., Wang, Y., Yang, S. & Tang, K. „An adaptive framework to tune the coordinate systems in nature-inspired optimization algorithms. *IEEE Trans. Cybernetics*. **49** (4), 1403–1416 (2019). 10.1109/TCYB.2018.2802912 29993876

[54] Braik, M., Hammouri, A., Atwan, J., Al-Betar, M. A. & Awadallah, M. A. „White Shark Optimizer: A novel bio-inspired meta-heuristic algorithm for global optimization problems, *Knowledge-Based Systems*, vol. 243, paper no. 108457, (2022).

[55] Houssein, E. H. et al. „An efficient discrete rat swarm optimizer for global optimization and feature selection in chemoinformatics, *Knowledge-Based Systems*, vol. 275, paper no. 110697, (2023).

[56] Kumar, S. et al. „Chaotic marine predators algorithm for global optimization of real-world engineering problems, *Knowledge-Based Systems*, vol. 261, paper no. 110192, (2023).

[57] Zhang, Q., Gao, H., Zhan, Z. H., Li, J. & Zhang, H. „Growth Optimizer: A powerful metaheuristic algorithm for solving continuous and discrete global optimization problems, *Knowledge-Based Systems*, vol. 261, paper no. 110206, (2023).

[58] Mostafa, R. R., Gaheen, M. A., ElAziz, M. A., Al-Betar, M. A. & Ewees, A. A. „An improved gorilla troops optimizer for global optimization problems and feature selection, *Knowledge-Based Systems*, vol. 269, paper no. 110462, (2023).

[59] Abdel-Salam, M., Alzahrani, A. I., Alblehai, F., Abu Zitar, R. & Abualigah, L. „An improved genghis khan optimizer based on enhanced solution quality strategy for global optimization and feature selection problems, *Knowledge-Based Systems*, paper no. 112237, (2024).

[60] Yang, X. S. „Nature-inspired optimization algorithms: challenges and open problems, *J. Comp. Sc.*, vol. 46, paper No. 101104, (2020).

[61] John, M. & Ammann, M. J. „Antenna optimization with a computationally efficient multiobjective evolutionary algorithm. *IEEE Trans. Ant Propag.* **57** (1), 260–263 (2009).

[62] Yang, X. S. *Nature-Inspired Optimization Algorithms* (Academic Press (Elsevier), 2021).

[63] Simon, D. *Evolutionary Optimization Algorithms* (John Wiley & Sons, Inc., 2013).

[64] Li, W., Zhang, Y. & Shi, X. „Advanced fruit fly optimization algorithm and its application to irregular subarray phased array antenna synthesis. *IEEE Access.* **7**, 165583–165596 (2019).

[65] Owoola, E. O., Xia, K., Wang, T., Umar, A. & Akindele, R. G. Pattern synthesis of uniform and sparse linear antenna array using mayfly algorithm. *IEEE Access.* **9**, 77954–77975 (2021).

[66] Koziel, S. & Ogurtsov, S. *Simulation-based Optimization of Antenna Arrays* (World Scientific, 2019).

[67] Queipo, N. V. et al. Surrogatebased analysis and optimization. *Progress Aero Sci.* **41** (1), 1–28 (2005).

[68] Koziel, S. & Pietrenko-Dabrowska, A. Rapid multi-objective optimization of antennas using nested kriging surrogates and single-fidelity EM simulation models. *Eng. Comp.* **37** (4), 1491–1512 (2019).

[69] Liu, J., Dong, H. & Wang, P. Multi-fidelity global optimization using a data-mining strategy for computationally intensive black-box problems, *Knowledge-Based Systems*, vol. 227, paper no. 107212, (2021).

[70] Hu, C., Zeng, S. & Li, C. A framework of global exploration and local exploitation using surrogates for expensive optimization, *Knowledge-Based Systems*, vol. 280, paper no. 111018, (2023).

[71] Dong, J., Qin, W. & Wang, M. Fast multi-objective optimization of multi-parameter antenna structures based on improved BPNN surrogate model, in IEEE Access, vol. 7, pp. 77692–77701, (2019).

[72] Mahouti, P., Belen, M. A., Calik, N. & Koziel, S. Computationally efficient surrogate-assisted design of pyramidal-shaped 3D reflectarray antennas, *IEEE Trans. Ant. Propag.*, Early view, (2022).

[73] de Villiers, D. I. L., Couckuyt, I. & Dhaene, T. Multi-objective optimization of reflector antennas using kriging and probability of improvement, *Int. Symp. Ant. Prop.*, pp. 985–986, San Diego, USA, (2017).

[74] Friedrichs, G. R., Elmansouri, M. A. & Filipovic, D. S. „A compact machine learning architecture for wideband amplitude-only direction finding. *IEEE Trans. Ant Propag.* **70** (7), 5189–5198 (2022).

[75] Jacobs, J. P. Characterization by Gaussian processes of finite substrate size effects on gain patterns of microstrip antennas. *IET Microwaves Ant Prop.* **10** (11), 1189–1195 (2016).

[76] Alzahed, A. M., Mikki, S. M. & Antar, Y. M. M. Nonlinear mutual coupling compensation operator design using a novel electromagnetic machine learning paradigm. *IEEE Ant Wirel. Prop. Lett.* **18** (5), 861–865 (2019).

[77] Tak, J., Kantemur, A., Sharma, Y. & Xin, H. A 3-D-printed W-band slotted waveguide array antenna optimized using machine learning. *IEEE Ant Wirel. Prop. Lett.* **17** (11), 2008–2012 (2018).

[78] Couckuyt, I., Declercq, F., Dhaene, T., Rogier, H. & Knockaert, L. Surrogate-based infill optimization applied to electromagnetic problems. *Int. J. RF Microw. Computt -Aided Eng.* **20** (5), 492–501 (2010).

[79] Liu, J., Han, Z. & Song, W. „Comparison of infill sampling criteria in kriging-based aerodynamic optimization, *28th Int. Congress of the Aeronautical Sciences*, pp. 1–10, Brisbane, Australia, 23–28 Sept., (2012).

[80] Torun, H. M. & Swaminathan, M. High-dimensional global optimization method for high-frequency electronic design. *IEEE Trans. Microw. Theory Techn*. **67** (6), 2128–2142 (2019).

[81] Cervantes-González, J. C. et al. Space mapping optimization of handset antennas considering EM effects of mobile phone components and human body. *Int. J. RF Microw. CAE*. **26** (2), 121–128 (2016).

[82] Koziel, S. & Unnsteinsson, S. D. Expedited design closure of antennas by means of trust-region-based adaptive response scaling. *IEEE Ant Wirel. Propag. Lett.* **17**(6), 1099–1103 (2018).

[83] Zhang, C., Feng, F., Gongal-Reddy, V., Zhang, Q. J. & Bandler, J. W. Cognition-driven formulation of space mapping for equal-ripple optimization of microwave filters. *IEEE Trans. Microw. Theory Techn*. **63** (7), 2154–2165 (2015).

[84] Lv, Z., Wang, L., Han, Z., Zhao, J. & Wang, W. Surrogate-assisted particle swarm optimization algorithm with Pareto active learning for expensive multi-objective optimization. *IEEE/CAA J. Automatica Sinica* **6**(3), 838–849 (2019).

[85] Taran, N., Ionel, D. M. & Dorrell, D. G. Two-level surrogate-assisted differential evolution multi-objective optimization of electric machines using 3-D FEA, *IEEE Trans. Magn.*, vol. 54, no. 11, paper 8107605, Nov. (2018).

[86] Koziel, S. Low-cost data-driven surrogate modeling of antenna structures by constrained sampling. *IEEE Antennas Wirel. Prop. Lett.* **16**, 461–464 (2017).

[87] Koziel, S. & Sigurdsson, A. T. Triangulation-based constrained surrogate modeling of antennas. *IEEE Trans. Ant Prop.* **66** (8), 4170–4179 (2018).

[88] Koziel, S. & Pietrenko-Dabrowska, A. Performance-based nested surrogate modeling of antenna input characteristics. *IEEE Trans. Ant Prop.* **67** (5), 2904–2912 (2019).

[89] Pietrenko-Dabrowska, A. & Koziel, S. Antenna modeling using variable-fidelity EM simulations and constrained co-kriging. *IEEE Access.* **8** (1), 91048–91056 (2020).

[90] Koziel, S. & Pietrenko-Dabrowska, A. *Performance-driven Surrogate Modeling of high-frequency Structures* (Springer, 2020).

[91] Koziel, S. & Pietrenko-Dabrowska, A. Constrained multi-objective optimization of compact microwave circuits by design triangulation and Pareto front interpolation. *Eur. J. Op Res.* **299** (1), 302–312 (2022).

[92] Pietrenko-Dabrowska, A., Koziel, S. & Al-Hasan, M. Expedited yield optimization of narrow- and multi-band antennas using performance-driven surrogates. *IEEE Access.*10.1109/ACCESS.2020.3013985 (2020).

[93] Koziel, S. Fast simulation-driven antenna design using response-feature surrogates. *Int. J. RF Micr CAE*. **25** (5), 394–402 (2015).

[94] Pietrenko-Dabrowska, A. & Koziel, S. „Generalized formulation of response features for reliable optimization of antenna structures, *IEEE Trans. Ant. Propag.*, Early View, (2021).

[95] Koziel, S. & Pietrenko-Dabrowska, A. Expedited feature-based quasi-global optimization of multi-band antennas with Jacobian variability tracking. *IEEE Access.* **8**, 83907–83915 (2020).

[96] Pietrenko-Dabrowska, A. & Koziel, S. Simulation-driven antenna modeling by means of response features and confined domains of reduced dimensionality. *IEEE Access.* **8**, 228942–228954 (2020).

[97] Koziel, S. & Bandler, J. W. A space-mapping approach to microwave device modeling exploiting fuzzy systems, *IEEE Trans. Microwave Theory and Tech.*, vol. 55, no. 12, pp. 2539–2547, Dec. (2007).

[98] Koziel, S. & Ogurtsov, S. *Antenna Design by simulation-driven Optimization. Surrogate-based Approach* (Springer, 2014).

[99] Pietrenko-Dabrowska, A. & Koziel, S. Surrogate modeling of impedance matching Transformers by means of variable-fidelity EM simulations and nested co-kriging. *Int. J. RF Microw. CAE*. **30** (8), e22268 (2020).

[100] Koziel, S., Ogurtsov, S., Bandler, J. W. & Cheng, Q. S. Reliable space mapping optimization integrated with EM-based adjoint sensitivities. *IEEE Trans. Microw. Theory Tech.* **61** (10), 3493–3502 (2013).

[101] Koziel, S., Pietrenko-Dabrowska, A. & Plotka, P. Reduced-cost microwave design closure by multi-resolution EM simulations and knowledge-based model management. *IEEE Access.* **9**, 116326–116337 (2021).

[102] Pietrenko-Dabrowska, A. & Koziel, S. Accelerated gradient-based optimization of antenna structures using multi-fidelity simulation models. *IEEE Trans. Ant Propag.* **69** (12), 8778–8789 (2021).

[103] Koziel, S. & Pietrenko-Dabrowska, A. Cost-efficient performance-driven modeling of multi-band antennas by variable-fidelity EM simulations and customized space mapping. *Int. J. Numer. Model.*10.1002/jnm.2778 (2020).

[104] Liu, B., Koziel, S. & Ali, N. SADEA-II: a generalized method for efficient global optimization of antenna design. *J. Comp. Des. Eng.* **4** (2), 86–97 (2017).

[105] Mahrokh, M. & Koziel, S. Explicit size-reduction of circularly polarized antennas through constrained optimization with penalty factor adaptation. *IEEE Access.* **9**, 132390–132396 (2021).

[106] Conn, A. R., Gould, N. I. M. & Toint, P. L. *Trust Region Methods* (MPS-SIAM Series on Optimization, 2000).

[107] Levy, H. & Lessman, F. *Finite Difference Equations* (Dover Publications Inc., 1992).

[108] Broyden, C. G. A class of methods for solving nonlinear simultaneous equations. *Math. Comp.* **19**, 577–593 (1965).

[109] Pietrenko-Dabrowska, A. & Koziel, S. Numerically efficient algorithm for compact microwave device optimization with flexible sensitivity updating scheme. *Int. J. RF Microw. CAE*10.1002/mmce.21714 (2019).

[110] Chen, Y. C., Chen, S. Y. & Hsu, P. Dual-band slot dipole antenna fed by a coplanar waveguide, *Proc. IEEE Antennas Propag. Soc. Int. Symp.*, Albuquerque, NM, USA, pp. 3589–3592, (2006).

[111] Pietrenko-Dabrowska, A. & Koziel, S. „Rapid variable-resolution parameter tuning of antenna structures using frequency-based regularization and sparse sensitivity updates, *IEEE Trans. Ant. Propag.*, Early View, (2022).

[112] Consul, P. Triple band gap coupled microstrip U-slotted patch antenna using L-slot DGS for wireless applications, *Communication, Control and Intelligent Systems (CCIS)*, Mathura, India, pp. 31–34, (2015).

[113] Farran, M. et al. Compact quasi-Yagi antenna with folded dipole fed by tapered integrated Balun. *Electron. Lett.* **52** (10), 789–790 (2016).

[114] Liu, F., Liu, Y., Han, F., Ban, Y. & Jay Guo, Y. Synthesis of large unequally spaced planar arrays utilizing differential evolution with new encoding mechanism and cauchy mutation. *IEEE Trans. Antennas Propag.* **68** (6), 4406–4416 (2020).

